# Vaccination coverage survey and seroprevalence among forcibly displaced Rohingya children, Cox's Bazar, Bangladesh, 2018: A cross-sectional study

**DOI:** 10.1371/journal.pmed.1003071

**Published:** 2020-03-31

**Authors:** Leora R. Feldstein, Sarah D. Bennett, Concepcion F. Estivariz, Gretchen M. Cooley, Lauren Weil, Mallick Masum Billah, M. Salim Uzzaman, Rajendra Bohara, Maya Vandenent, Jucy Merina Adhikari, Eva Leidman, Mainul Hasan, Saifuddin Akhtar, Andreas Hasman, Laura Conklin, Daniel Ehlman, A. Alamgir, Meerjady Sabrina Flora

**Affiliations:** 1 Epidemic Intelligence Service, Centers for Disease Control and Prevention, Atlanta, Georgia, United States of America; 2 Global Immunization Division, Centers for Disease Control and Prevention, Atlanta, Georgia, United States of America; 3 Division of Parasitic Diseases and Malaria, Centers for Disease Control and Prevention, Atlanta, Georgia, United States of America; 4 Division of Bacterial Diseases, Centers for Disease Control and Prevention, Atlanta, Georgia, United States of America; 5 Institute of Epidemiology, Disease Control and Research, Dhaka, Bangladesh; 6 World Health Organization, Dhaka, Bangladesh; 7 United Nations Children’s Fund, Dhaka, Bangladesh; 8 Division of Global Health Protection, Centers for Disease Control and Prevention, Atlanta, Georgia, United States of America; 9 United Nations Children’s Fund, Regional Office for South Asia, Kathmandu, Nepal; International Organization for Migration, SRI LANKA

## Abstract

**Background:**

During August 2017–January 2018, more than 700,000 forcibly displaced Rohingyas crossed into Cox’s Bazar, Bangladesh. In response to measles and diphtheria cases, first documented in September and November 2017, respectively, vaccination campaigns targeting children <15 years old were mobilized during September 2017–March 2018. However, in a rapidly evolving emergency situation, poor sanitation, malnutrition, overcrowding, and lack of access to safe water and healthcare can increase susceptibility to infectious diseases, particularly among children. We aimed to estimate population immunity to vaccine-preventable diseases (VPDs) after vaccination activities in the camps to identify any remaining immunity gaps among Rohingya children.

**Methods and findings:**

We conducted a cross-sectional serologic and vaccination coverage survey in Nayapara Registered Refugee Camp (“Nayapara”) and makeshift settlements (MSs) April 28, 2018 to May 31, 2018, among 930 children aged 6 months to 14 years. MSs are informal, self-settled areas with a population of more than 850,000, the majority of whom arrived after August 2017, whereas Nayapara is a registered camp and has better infrastructure than MSs, including provision of routine immunization services. Households were identified using simple random sampling (SRS) in Nayapara and multistage cluster sampling in MSs (because household lists were unavailable). Dried blood spots (DBSs) were collected to estimate seroprotection against measles, rubella, diphtheria, and tetanus, using Luminex multiplex bead assay (MBA). Caregiver interviews assessed vaccination campaign participation using vaccination card or recall. In Nayapara, 273 children aged 1 to 6 years participated; 46% were female and 88% were registered refugees. In MSs, 358 children aged 1 to 6 years and 299 children aged 7 to 14 years participated; 48% of all children in MSs were female, and none were registered refugees. In Nayapara, estimated seroprotection among 1- to 6-year-olds was high for measles, rubella, diphtheria, and tetanus (91%–98%; 95% confidence interval [CI] 87%–99%); children >6 years were not assessed. In MSs, measles seroprotection was similarly high among 1- to 6-year-olds and 7- to 14-year-olds (91% [95% CI 86%–94%] and 99% [95% CI 96%–100%], respectively, *p* < 0.001). Rubella and diphtheria seroprotection in MSs were significantly lower among 1- to 6-year-olds (84% [95% CI 79%–88%] and 63% [95% CI 56%–70%]) compared to 7- to 14-year-olds (96% [95% CI 90%–98%] and 77% [95% CI 69%–84%]) (*p* < 0.001). Tetanus seroprevalence was similar among 1- to 6-year-olds and 7- to 14-year-olds (76% [95% CI 69%–81%] and 84% [95% CI 77%–89%], respectively; *p* = 0.07). Vaccination campaign coverage was consistent with seroprotection in both camps. However, nonresponse, the main limitation of the study, may have biased the seroprotection and campaign coverage results.

**Conclusions:**

In this study, we observed that despite multiple vaccination campaigns, immunity gaps exist among children in MSs, particularly for diphtheria, which requires serial vaccinations to achieve maximum protection. Therefore, an additional tetanus-diphtheria campaign may be warranted in MSs to address these remaining immunity gaps. Rapid scale-up and strengthening of routine immunization services to reach children and to deliver missed doses to older children is also critically needed to close immunity gaps and prevent future outbreaks.

## Abstract 2

In a cross-sectional study, Leora R. Feldstein and colleagues investigate vaccination coverage and seroprevalence among forcibly-displaced Rohingya children in Cox’s Bazar, Bangladesh in 2018.

## Author summary

**Why was this study done?:**

- Rohingyas living in camps and makeshift settlements (MSs) are at risk of outbreaks of vaccine-preventable diseases (VPDs).
- Ongoing outbreaks of measles and diphtheria, despite vaccination campaigns, suggest gaps in immunity for some children.
- We conducted this survey among Rohingya children to better understand these immunity gaps and risk for ongoing and future outbreaks of VPDs.

**What did the researchers do and find?:**

- We conducted a cross-sectional serologic and vaccine coverage survey in 2 camps in April and May 2018.
- The youngest children living in the MSs, who are more recent arrivals from Myanmar, had low levels of seroprotection for rubella (84% of children), diphtheria (63%), and tetanus (76%).
- Seroprotection for the youngest children living in Nayapara registered camp was high for measles, rubella, diphtheria, and tetanus (91%–98% of children).

**What do these findings mean?:**

- These findings suggest that despite vaccination campaigns, immunity gaps remain for VPDs, especially among children who are more recent arrivals from Myanmar and now live in MSs.
- Remaining immunity gaps may increase the risk of outbreaks of VPDs.
- An additional diphtheria-tetanus containing vaccine (DTCV) campaign may be beneficial to address the very low seroprotection levels identified among children living in the MSs.
- Rapid scale-up of routine immunization services is necessary to close remaining immunity gaps among children and those that have missed campaign opportunities.
- Nonresponse, the main limitation of this study, may have biased the seroprotection and campaign coverage results.

## Introduction

During August 2017–January 2018, more than 700,000 forcibly displaced Rohingyas fled from violence in Rakhine State, Myanmar, to Cox’s Bazar, Bangladesh, joining more than 200,000 Rohingyas already living in refugee camps in the region [1]. Complex public health issues of concern—including poor sanitation, malnutrition, overcrowding, and lack of access to safe water and healthcare—increase susceptibility to infectious diseases, particularly among children [2]. World Health Organization (WHO) guidelines emphasize the importance of using multiple modes of vaccination delivery in complex emergencies, including campaigns, vaccination upon entry, and routine immunization services. Prior to August 2017, routine services were available at a subset of health facilities in registered camps; however, these facilities had limited reach and lacked resources and infrastructure to serve new arrivals [3].

In September and November 2017, the first suspected measles and diphtheria cases were reported in Cox’s Bazar [4]. In addition to the delivery of routine services, immunization campaigns are crucial for controlling outbreaks of vaccine-preventable diseases (VPDs). Two doses of measles-rubella (MR) vaccine and three doses of diphtheria-tetanus containing vaccine (DTCV) are recommended for providing immune protection against these VPDs [5, 6]. Therefore, 2 rounds of MR and 3 rounds of DTCV campaigns as well as 2 campaign rounds of oral cholera vaccine (OCV) (as a preventive measure) were conducted in Cox’s Bazar during September 2017–March 2018, primarily targeting children younger than 15 years (S1 Table). Despite these efforts, outbreaks of measles and diphtheria continued in registered camps and makeshift settlements (MSs). As of August 28, 2018, more than 3,000 suspected measles cases and 8,000 suspected diphtheria cases had been reported among Rohingyas in Cox’s Bazar [4, 7].

Little is known about vaccination coverage among Rohingya children or their access to routine immunizations or vaccination campaigns prior to their arrival in Cox’s Bazar. During the September–December 2017 vaccination campaigns in the refugee camps and MSs, vaccination teams observed some hesitancy among Rohingyas to participate in vaccination campaigns. As a result, focus group discussions and key informant interviews with caregivers of children living in the MSs, camp leaders, religious leaders, and teachers were conducted to identify potential barriers to participation in the vaccination campaigns. Findings from the assessment highlighted several barriers, including beliefs about vaccination causing people to become Christian, concerns about multiple vaccinations given on the same day, worries about vaccination side effects, and lack of sensitivity to cultural gender norms at vaccination sites [8].

As a follow-up to the qualitative assessment, we conducted a cross-sectional serologic and vaccination coverage survey in 2 areas of Cox’s Bazar: Nayapara Registered Refugee Camp (henceforth referred to as Nayapara) and the MSs in April–May 2018. Nayapara, established in 1992, is a registered refugee camp managed by the United Nations High Commissioner for Refugees (UNHCR) with an estimated population just under 23,000 [9]. MSs are informal, self-settled areas with a population of more than 850,000, the majority of whom arrived after August 2017 [9]. Because of its longstanding status, Nayapara has better infrastructure than MSs, including provision of routine immunization services, whereas MSs were—until recently—uninhabited land and therefore have limited facilities providing routine immunizations [10]. We aimed to estimate population immunity to VPDs after vaccination activities in Nayapara and MSs to identify any remaining immunity gaps specifically among Rohingya children 6 months to 14 years.

## Methods

This study is reported as per the Strengthening the Reporting of Observational Studies in Epidemiology (STROBE) guideline (S1 STROBE Checklist). A prospective protocol and analysis plan were used in designing the study (S1 Protocol).

### Study design

The vaccine coverage and seroprevalence survey among Rohingya children was conducted in Nayapara and MSs in conjunction with an Emergency Nutrition Assessment that was concurrently conducted by the Nutrition Sector (led by Action Against Hunger) [11]. Sampling methodology differed between Nayapara and MSs because of differences in population size and how the two areas are organized. In Nayapara, households were selected using simple random sampling (SRS) of household lists updated the week preceding data collection. In MSs, where household lists were not available, cluster sampling design was used, wherein camps were subdivided into existing blocks and sub-blocks (median size = 108 households, range 26–970). Fifty-five sub-blocks (clusters) were selected from a complete list of sub-blocks (IOM Displacement Tracking Matrix, round 12, September 4, 2018) using population proportional to size. Enumeration of households in selected clusters took place in the 9 days preceding the start of data collection. Thirteen households were selected from each cluster’s enumeration list using SRS. Within each selected household, one child 6 months to 6 years and one child 7 to 14 years were selected among age-eligible children using a random number generator (SI Appendix). The target sample size for the serologic survey was 270 children in Nayapara and 706 in MSs (SI Appendix). There were no exclusions of households because of inaccessibility in either camp.

Selected children were enrolled in the survey, which included a questionnaire and collection of dried blood spots (DBSs) for serologic analysis. Only children 1 to 14 years old were eligible to participate in the serologic component of the survey in MSs. Children <1 year were excluded due to concerns about collecting sufficient blood quantity. Data collection in Nayapara took place during Ramadan, and many older children were expected to fast; as a result, only children 1 to 6 years old were eligible for both the serologic and questionnaire component of the survey. The following analysis includes vaccine coverage and serologic data from children 1 to 14 years old in MSs and 1 to 6 years old in Nayapara.

Interviews were conducted in or near homes with available caregiver(s) for each selected child; these included a vaccine coverage questionnaire to assess vaccines received during the campaigns, prior to arrival in Bangladesh and at the border, as well as barriers to receiving vaccination during the most recent vaccination campaign. Available vaccination cards were reviewed to record documented vaccinations received; caregiver recall was also accepted. DBSs were collected to test for presence of antibodies to diphtheria, tetanus, measles, and rubella by microneutralization and Luminex multiplex bead assay (MBA) (S1 Appendix). Because resources were limited, testing for vibriocidal antibodies (a marker of protection against cholera) was not done. Efforts were made to return to households where residents were absent or whose children were absent at the time of the initial visit and DBS collection.

The vaccine coverage and seroprevalence survey among Rohingya children was also planned in Kutupalong Refugee camp; however, data collection was interrupted early. Teams encountered high rates of refusals due to fears of loss of benefits, of loss of refugee status, and of relocation. Data collection was discontinued given concerns about representativeness due to high nonresponse rate caused by high numbers of systematic refusals, and we chose not to report these results.

### Data collection

Data were collected on electronic tablets using KoBoCollect software (version 1.4.8) and uploaded daily to a server to enable remote monitoring of data quality. The questionnaire was standardized in English and Bangla, translated into Rohingya (an oral language) through group consensus, and delivered by speakers of Rohingya or Chittagonian, a language similar to Rohingya (S1 Questionnaire).

### Specimen collection, storage, and transport

Using 1.8-mm lancets, up to 4 drops of blood were collected from participants and applied to Whatman 903 Protein Saver Cards. The cards were kept on drying racks inside plastic boxes to allow them to dry for at least 4 hours. The cards were subsequently stored in individual, sealed plastic bags with a 5 g silica gel desiccant sachet to protect against humidity and a humidity-monitoring card for up to 2 weeks. Humidity monitoring cards were checked daily. DBS samples were transported to Centers for Disease Control and Prevention (CDC) laboratories in the United States, where they were stored at −20°C until testing.

### Statistical analysis

Estimation of seroprotection and vaccination coverage (using vaccination card and caregiver recall) and 95% confidence intervals (CIs) was done using standard (Nayapara) or complex survey procedures accounting for cluster design and sampling weights (MS) [12]. χ^2^ tests were used to examine differences in immunity by age group, sex, and Bacillus Calmette-Guérin (BCG) vaccination status in MSs. Only children enrolled in both the vaccination coverage and serologic survey were included in the analysis. All analyses were completed in STATA 14. Statistical significance was defined as *p* < 0.05.

### Ethics approval and consent

The survey was approved by the Bangladesh Insititute of Epidemiology, Disease Control, and Research institutional review board. The US CDC’s Human Subjects Office considered the survey an epidemic disease control activity. Participation was voluntary, and the objectives, length of survey, and risks/benefits of participation were explained to individuals prior to enrollment. The caregiver’s verbal informed consent was obtained.

## Results

### Enrollment

Study teams visited 715 households in MSs; questionnaires were completed, and adequate DBS samples were collected from 657 (83%) of 789 eligible children (Table 1). Of the 524 households visited in Nayapara, questionnaires were completed, and adequate DBS samples were collected from 273 (97%) of 281 eligible children (only children 1–6 years old).

**Table 1:** Eligibility and enrollment of participants, serologic and vaccination coverage survey among Rohingya children, by geographic area, Cox’s Bazar, Bangladesh 2018.

	MS	Nayapara
**Number of households visited**	715	524
Number enrolled^*^	675 (94%)	483 (92%)
**Number of children enrolled**	829	638
**Number eligible for DBS collection**^†^	789 (95%)	281 (44%)
**Number with adequate DBS collected**	657 (83%)	273 (97%)
**Number without DBS**	132 (17%)	8 (3%)
Not at home	92 (70%)	6 (75%)
Parent refused	3 (2%)	0 (0%)
Child refused	15 (11%)	1 (13%)
Insufficient blood draw	20 (15%)	1 (13%)
Other reason (e.g., child visibly sick)	2 (2%)	0 (0%)

*One household in the MSs refused participation; 39 households were not at home at the time of the survey.

^†^Children 6–11 months of age were ineligible for DBS sample collection; DBS samples were not collected from children 7–14 years old in Nayapara because of Ramadan.

**Abbreviations:** DBS, dried blood spot; MS, makeshift settlement

### Demographic characteristics and historical vaccination status

The majority of children in Nayapara were registered refugees (88%), born in Bangladesh (89%), and had received BCG vaccination as confirmed by injection-site scar or vaccination card (84%) (Table 2). In contrast, most of the children in MSs were recent arrivals (93%–95%), few were born in Bangladesh (3%), and none were registered refugees. Children 7 to 14 years old in MSs had evidence of BCG vaccination (72%) more commonly than children 1 to 6 years old (51%). For all children, vaccination card retention was low: 14% in Nayapara and 20%–21% in MSs. Among children eligible to receive vaccinations in Myanmar (prior to arrival in Bangladesh), injections were reportedly received in Myanmar by 53% of children 1 to 6 years old in Nayapara and in 66% of 1- to 6-year-olds and 87% of 7- to 14-year-olds in MSs. Among children eligible to receive vaccinations upon entry into Bangladesh, injections were reportedly received among 3% (*n* = 1/32) in Nayapara and among 1% (*n* = 3/374) of 1-to 6-year-olds and 2% (*n* = 5/289) of 7- to 14-year-olds in MSs. Mean household size and percentage of female children enrolled were similar across geographic areas.

**Table 2:** Demographic characteristics and historical vaccination status,* by geographic area and age group, serologic and vaccination coverage survey among Rohingya children, Cox’s Bazar, Bangladesh, 2018.

	MS		Nayapara
	**1–6 years (*N* = 358)**	**7–14 years (*N* = 299)**	**1–6 years (*N* = 273)**
**Household size, mean (range)**	5.6 (2–16)	6.0 (2–16)	5.9 (2–14)
**Registered refugees**	0 (0%)	0 (0%)	240 (88%)
**Arrival after July 2017**	339 (95%)	277 (93%)	31 (11%)
**Child born in the camp (in Bangladesh)**	11 (3%)	10 (3%)	241 (89%)
**Sex (female)**	170 (47%)	147 (49%)	126 (46%)
**Evidence of BCG vaccination (scar or card)**	171 (51%)	211 (72%)	228 (84%)
**Reported receiving injections in Myanmar**^†^	229 (66%)	250 (87%)	17 (53%)
**Retained at least 1 vaccination card from campaigns**	73 (20%)	63 (21%)	39 (14%)
**Received vaccination upon entry to Bangladesh**^†^	3 (1%)	5 (2%)	1 (3%)

*Among children who provided an adequate DBS sample.

^†^Denominator is children who were not born in Bangladesh.

**Abbreviations:** BCG, Bacillus Calmette-Guérin; DBS, dried blood spot; MS, makeshift settlement

### Measles and rubella campaign coverage and seroprotection

The two MR vaccination campaigns took place during September 16, 2017 to October 3, 2017 and during November 18, 2017 to December 5, 2017 (S1 Table). In MSs, MR campaign coverage estimates among children 1 to 6 years old were 90% (95% CI 85%–94%) for at least one dose and 66% (95% CI 57%–75%) for two doses compared to 82% (95% CI 71%–90%) and 65% (95% CI 53%–75%) among 7- to 14-year-olds (Table 3). In Nayapara, among children 1 to 6 years old, vaccination campaign coverage for at least one dose of MR vaccine was 94% (95% CI 90%–96%) and 84% (95% CI 79%–87%) for two doses.

**Table 3:** Vaccination campaign coverage,* by geographic area and age group, serologic and vaccination coverage survey among Rohingya children, Cox’s Bazar, Bangladesh, 2018.

Campaign	MS		Nayapara
** **	**1–6 years (*n* = 338)**^†^ **(95% CI)**	**7–14 years (*n* = 292)**^†^ **(95% CI)**	**1–6 years (*n* = 268)**^†^ **(95% CI)**
MR (at least 1 dose)	90% (85%–94%)	82% (71%–90%)	94% (90%–96%)
MR (2 doses)	66% (57%–75%)	65% (53%–75%)	84% (79%–87%)
DTCV (at least 1 dose)	92% (84%–96%)	90% (81%–95%)	98% (95%–99%)
DTCV (at least 2 doses)	88% (80%–93%)	84% (74%–90%)	96% (92%–98%)
DTCV (3 doses)	68% (60%–76%)	76% (67%–83%)	88% (83%–92%)
OCV (at least 1 dose)	92% (84%–96%)	-	99% (96%–100%)
OCV (2 doses)	90% (81%–95%)	-	98% (96%–99%)

*Among children who provided an adequate DBS sample; coverage was estimated using vaccination card and caregiver recall.

^†^Denominators varied slightly for each campaign depending on eligibility for campaign participation (based upon date of arrival and child age).

**Abbreviations:** DBS, dried blood spot; DTCV, diphtheria-tetanus containing vaccine; MR, measles-rubella vaccine; MS, makeshift settlement; OCV, oral cholera vaccine.

**Fig 1:**
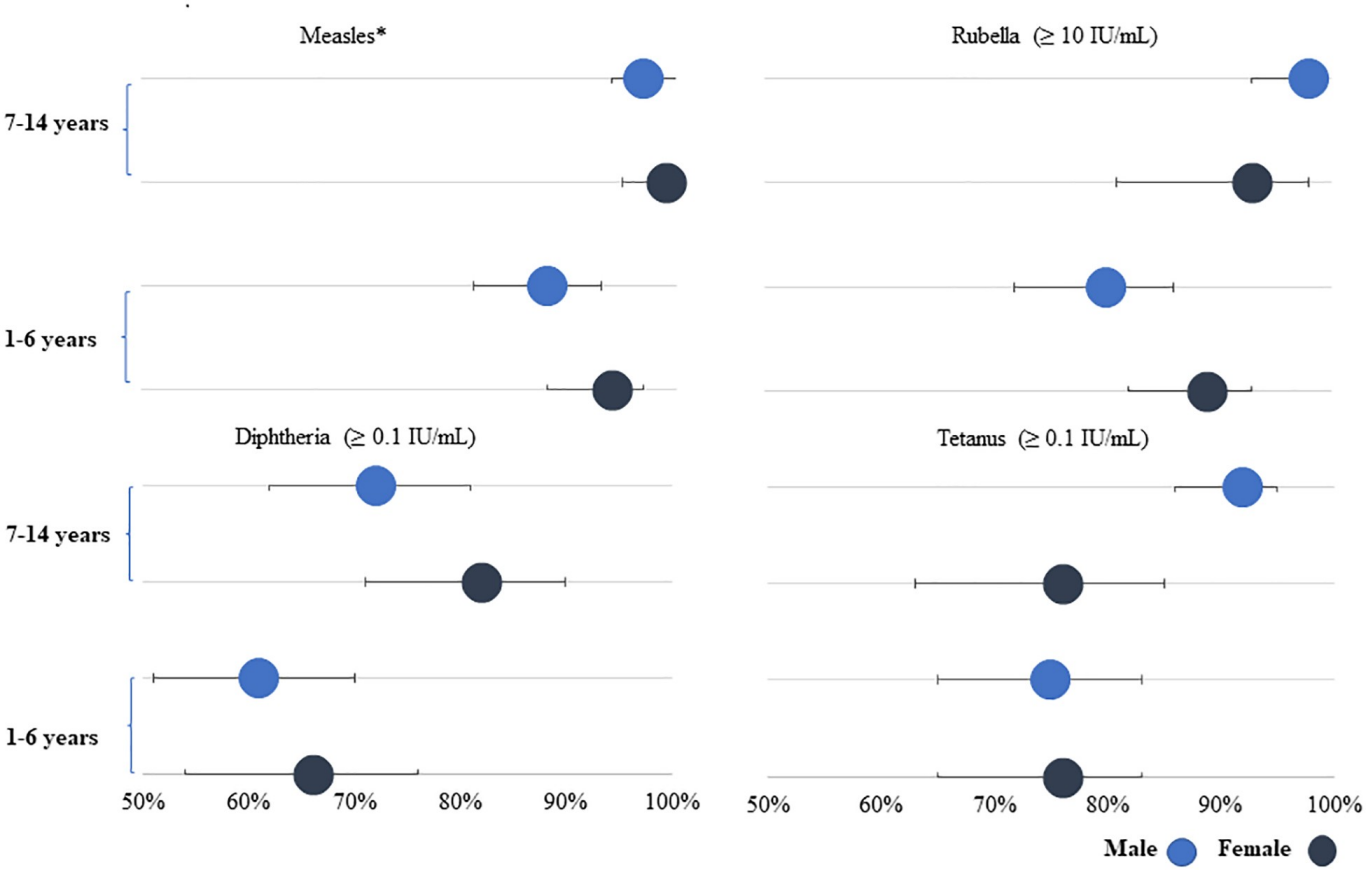
Percentage of children seroprotected based upon predefined antibody level cutoffs by antigen and sex, MSs, serologic and vaccination coverage survey among Rohingya children, Cox’s Bazar, Bangladesh, 2018. Children 1–6 years old: *n* = 358; children 7–14 years old: *n* = 299. *Measles virus nucleoprotein cutoff was determined empirically at the CDC, calculated by ROC with samples characterized with PRNT, where samples >120 mIU/mL were considered positive. CDC, Centers for Disease Control and Prevention; IU, international units; MS, makeshift settlement; PRNT, plaque reduction neutralization test; ROC, receiver operating characteristic.

The estimated seroprotection of children aged 1 to 6 years old in MSs was 91% (95% CI 86%–94%) for measles and 84% (95% CI 79%–88%) for rubella, compared to 99% (95% CI 96%–100%) and 96% (95% CI 90%–98%), respectively, among children 7 to 14 years old (*p* < 0.001) (Table 4). In Nayapara, the estimated seroprotection of children 1 to 6 years old was 97% (95% CI 94%–99%) for measles and 98% (95% CI 95%–99%) for rubella. Measles and rubella seroprotection in MSs were similar among females and males (Fig 1). In Nayapara, seroprotection for measles was similar among females and males and across age categories.

**Table 4:** Percentage of children seroprotected based upon predefined antibody level cutoffs for measles, rubella, tetanus, and diphtheria, by geographic area and age group, serologic and vaccination coverage survey among Rohingya children, Cox’s Bazar, Bangladesh, 2018.

	MS		Nayapara
	**1–6 years (*n* = 358)** (95% CI)	**7–14 years (*n* = 299)** (95% CI)	**1–6 years (*n* = 273)** (95% CI)
Measles^*^	91% (86%–94%)^†^	99% (96%–100%)^†^	97% (94%–99%)
**Rubella (≥10 IU/mL)**	84% (79%–88%)^†^	96% (90%–98%)^†^	98% (95%–99%)
**Diphtheria**			
≥0.01 IU/mL	88% (83%–92%)	93% (85%–96%)	99% (97%–99%)
≥0.1 IU/mL	63% (56%–70%)^†^	77% (69%–84%)^†^	91% (87%–94%)
≥1.0 IU/mL	17% (13%–21%)^†^	30% (25%–35%)^†^	27% (22%–33%)
**Tetanus**			
≥0.01 IU/mL	91% (86%–95%)	96% (92%–98%)	98% (96%–99%)
≥0.1 IU/mL	76% (69%–81%)	84% (77%–89%)	97% (95%–99%)
≥1.0 IU/mL	47% (40%–55%)^†^	69% (62%–76%)^†^	79% (74%–83%)

*Measles virus nucleoprotein cutoff was determined empirically at the CDC, calculated by ROC with samples characterized with PRNT, where samples >120 mIU/mL were considered positive.

^†^Comparison between age groups in MSs, significant at the *p* < 0.05 level using χ^2^ tests.

**Abbreviations:** CDC, Centers for Disease Control and Prevention; CI, confidence interval; IU, international units; MS, makeshift settlement; PRNT, plaque reduction neutralization test; ROC, receiver operating characteristic

### Tetanus and diphtheria campaign coverage

The 3 DTCV vaccination campaigns took place during December 12, 2017, to December 31, 2017; January 27, 2018, to February 10, 2018; and March 10, 2018, to March 25, 2018 (S1 Table). In MSs, DTCV campaign coverage estimates among children 1 to 6 years old were 92% (95% CI 84%–96%) for at least one dose and 88% (95% CI 80%–93%) for at least two doses, compared to 90% (95% CI 81%–95%) for at least one dose and 84% (95% CI 74%–90%) for at least two doses among 7- to 14-year-olds (Table 3). In Nayapara, vaccination campaign coverage for at least one dose of DTCV was 98% (95% CI 95%–99%) and 95% (95% CI 92%–98%) for at least two doses (Table 3).

**Fig 2:**
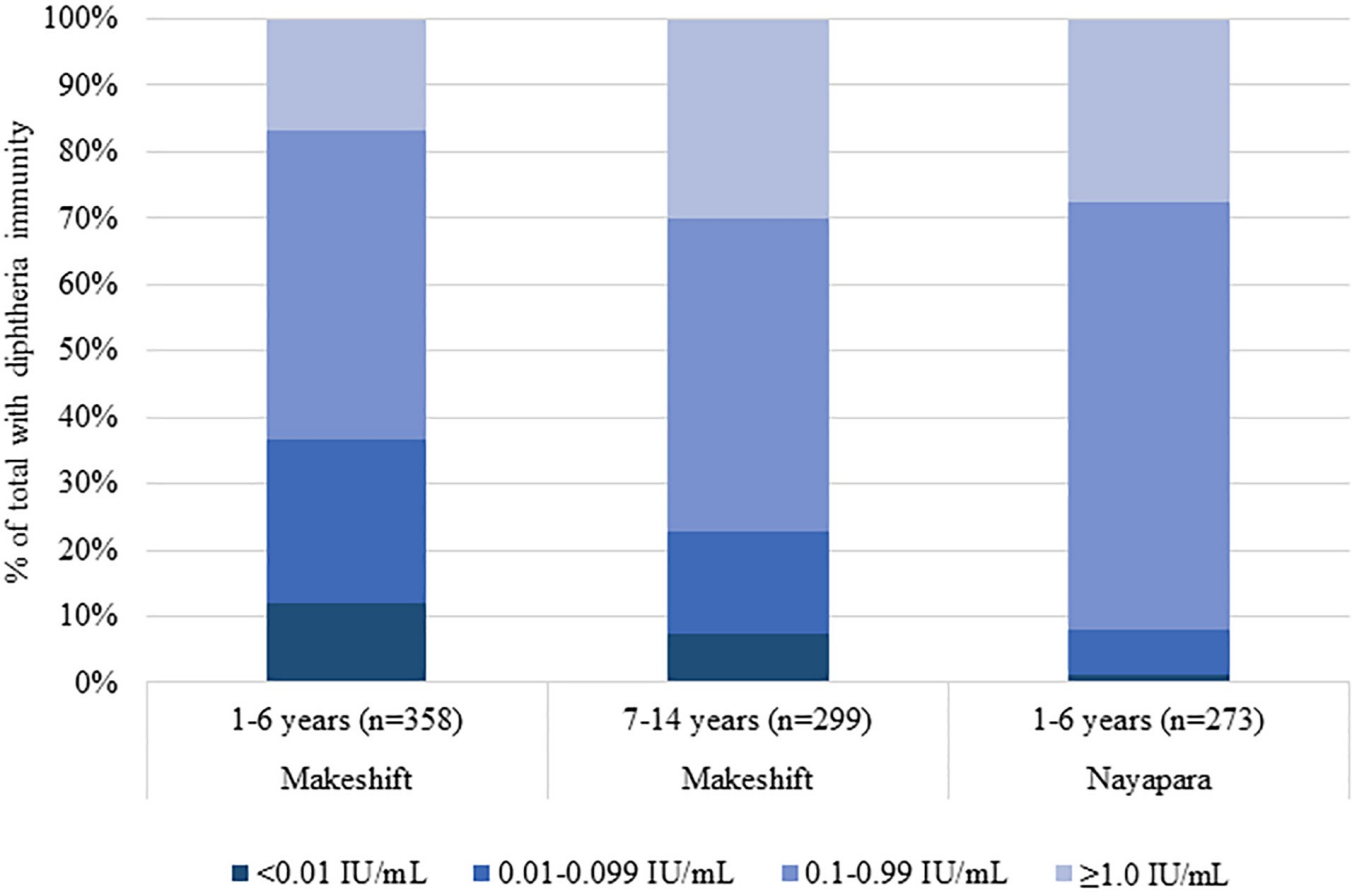
Diphtheria antibody levels, by geographic area and age group, serologic and vaccination coverage survey among Rohingya children, Cox’s Bazar, Bangladesh, 2018. IU, international units.

**Fig 3:**
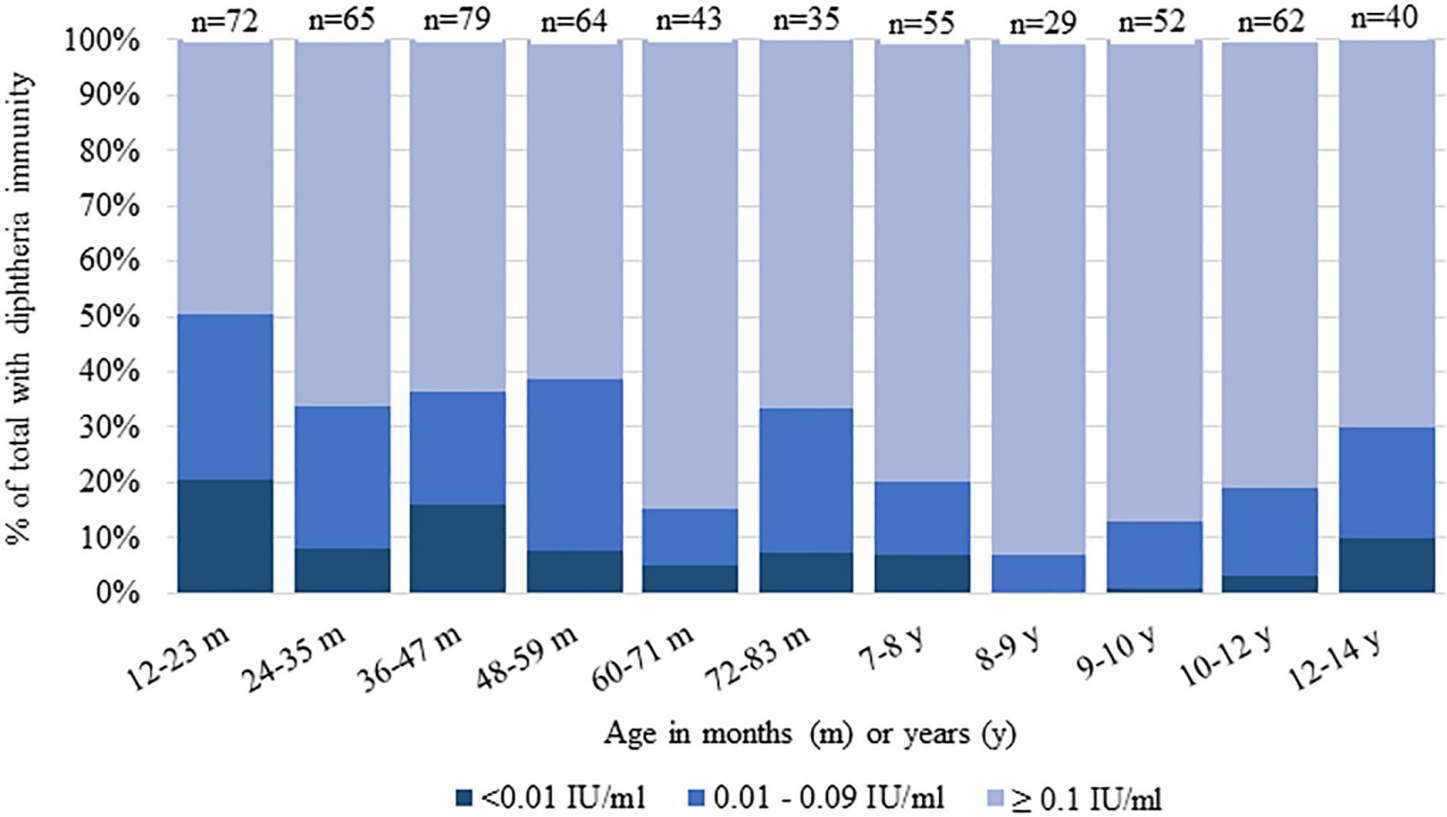
Diphtheria antibody levels in the MSs by age category, serologic and vaccination coverage survey among Rohingya children, Cox’s Bazar, Bangladesh, 2018. IU, international units; MS, makeshift settlement.

Estimated seroprotection against diphtheria at the ≥0.1 IU/mL cutoff among children aged 1 to 6 years in MSs was 63% (95% CI 56%–70%) compared to 77% (95% CI 69%–84%) among 7- to 14-year-olds (*p* < 0.001) (Table 4, Fig 2). In this survey, children 12 to 23 months (*n* = 72) in MSs were the least protected against diphtheria, with 20% having no protection (<0.01 IU/mL) and 30% having minimal protection (0.01–0.09 IU/mL) (Fig 3). Diphtheria seroprotection in MSs was similar among females and males (Fig 1). Unweighted diphtheria seroprotection varied by cluster ranging from 25% to 100%, with 5 of the 55 clusters showing <50% seroprotection. These 5 clusters were spread throughout MSs, and none was adjacent to another.

Estimated seroprotection against tetanus at the ≥0.1 IU/mL cutoff among children aged 1 to 6 years in MSs was 76% (95% CI 69%–81%), compared to 84% (95% CI 77%–89%) among children 7 to 14 years old (*p* = 0.07, Table 4). Children aged 1 to 6 years with evidence of BCG vaccination were more likely to be seroprotected against diphtheria 78% (95% CI 70%–85%) and tetanus 86% (95% CI 79%–91%) than those who had no evidence of BCG vaccination (50% [95% CI 40%–61%] and 66% [95% CI 54%–76%], respectively [*p* < 0.001]).

**Fig 4:**
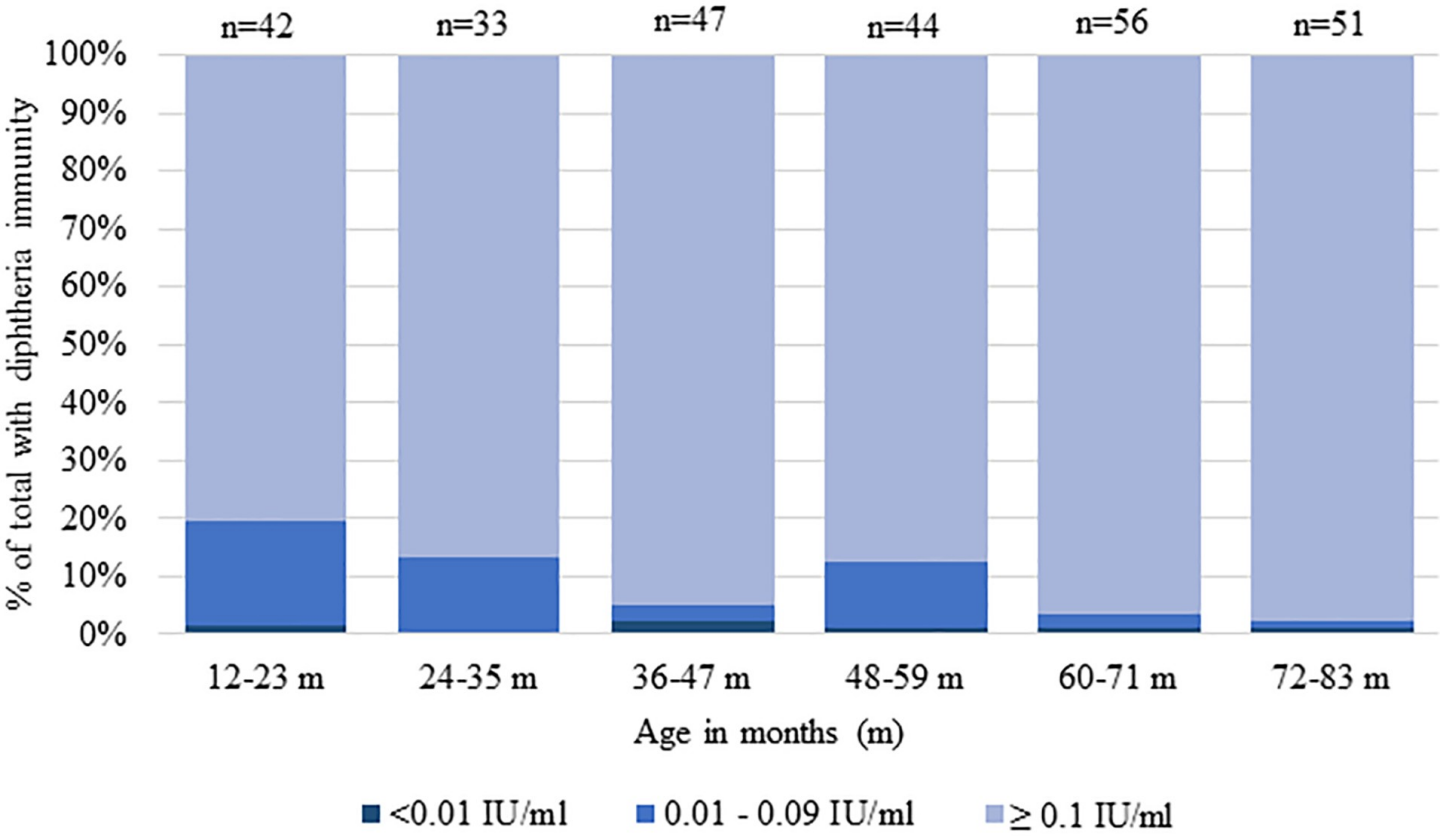
Diphtheria antibody levels in Nayapara by age category, serologic and vaccination coverage survey, Cox’s Bazar among Rohingya children, Bangladesh, 2018. IU, international units.

In Nayapara, estimated seroprotection against diphtheria and tetanus at the ≥0.1 IU/mL cutoff among children 1 to 6 years old was 91% (95% CI 87%–94%) and 97% (95% CI 95%–99%), respectively (Table 4). Seroprotection for tetanus and diphtheria was similar among females and males and varied only slightly by 12-month age category for diphtheria (Fig 4).

### Cholera campaign coverage

Two OCV campaigns took place during October 10, 2017, to October 18, 2017, and November 4, 2017, to November 9, 2017 (S1 Table). In MSs, OCV campaign coverage estimates among children 1 to 6 years old were 92% (95% CI 84%–96%) for at least one dose and 90% (95% CI 81%–95%) for two doses. Children aged 7 to 14 years were not eligible for either OCV campaigns due to resource constraints but were eligible for subsequent OCV campaigns after this survey was conducted. In Nayapara, among children 1 to 6 years old, vaccination campaign coverage for at least one dose of OCV was 99% (95% CI 96%–100%) and 98% (95% CI 96%–99%) for two doses.

### Reasons for not vaccinating

In MSs, 18% (*n* = 115/657) of caregivers reported not vaccinating their child during the third round of the DTCV campaign, compared to 6% (*n* = 16/273) in Nayapara. Among all surveyed populations (by age and camp), the top 3 reasons for nonvaccination were consistent: caregiver was unaware of the campaign (39%), child was not available (34%), and child was afraid of needles/pain (33%) (S2 Table). In MSs, 12- to 23-month-olds (the group least protected against diphtheria) represented 20% of the sample of 1- to 6-year-olds but constituted 35% (*n* = 23/66) of all children aged 1 to 6 years whose mothers/caretakers chose not to have them vaccinated during the third DTCV campaign.

## Discussion

These findings suggest that although multiple rounds of MR and DTCV vaccination campaigns in MSs and Nayapara likely improved immunity to VPDs among children 1 to 14 years old, immunity gaps remain in both camps, particularly for diphtheria in MSs among younger children. To a lesser extent, immunity gaps also remain for tetanus in both age groups and for rubella in the younger age group in MSs. The presence of these immunity gaps is supported by continued reporting of suspected measles and diphtheria cases. Based on results from the survey, very few were vaccinated at the border, and vaccine uptake during campaigns may have been hindered by caregivers’ lack of awareness of campaigns and unavailability of children during campaigns because of school, daycare, or labor. Prior to this assessment, there was limited information about vaccination coverage among Rohingya children and their access to routine immunization services and vaccination campaigns before their arrival to Cox’s Bazar. This serosurvey and vaccination coverage survey provides a timely evaluation of remaining immunity gaps for VPDs among this population.

Significant differences in seroprotection were observed comparing older and younger children among the recently arrived; seroprotection for both rubella and diphtheria was higher among 7- to 14-year-olds compared to 1- to 6-year-olds in MSs. Additionally, a larger proportion of 7- to 14-year-olds born in Myanmar reported receipt of injections in Myanmar and proof of BCG vaccination compared to 1- to 6-year-olds. This difference may be attributable, in part, to the declining access to health services in Myanmar in recent years due to violence against the Rohingya population in Rakhine State [13]. Routine immunizations in Myanmar are usually administered within the first 2 years of life, including BCG, which is given within 6 weeks of birth and can be used as a marker for access to and utilization of routine services [14]. Thus, children 7 to 14 years old in MSs, the majority of whom were born in Myanmar, may have been more likely to receive all recommended vaccines in the national schedule than children 1 to 6 years old, who were born more recently during the violence and political unrest. It is also possible that children 7 to 14 years old had higher seroprotection simply because of increased exposure to VPDs and therefore more natural immunity than children 1 to 6 years old.

In MSs, children 1 to 6 years old with evidence of BCG vaccination were more likely to be seroprotected against diphtheria, which is not surprising because children who received BCG vaccine likely had better access to and acceptance of other vaccines [15, 16].

Seroprotection increased with the number of MR and DTCV doses received during campaigns in MSs. Although campaign coverage from recall and card was consistent with seroprotection for most antigens, MR campaign coverage for at least one dose in MSs among 7- to 14-year-olds (82%) was lower than measles seroprotection (99%) and rubella seroprotection (96%). This difference might be explained by protection obtained through exposure to the ongoing transmission of measles virus (>3,000 suspected measles cases during September 2017–July 2018) and potentially rubella virus, previous outbreaks of measles in Myanmar, and MR vaccination in Myanmar prior to arriving in Bangladesh [7]. Conversely, DTCV campaign coverage for at least two doses among 1- to 6-year-olds in MSs (88%) was higher than diphtheria seroprotection (63%) and tetanus seroprotection (76%). Seroprotection may have been lower than expected because the third round of the DTCV campaign may have been conducted too soon after the second campaign (approximately 4 weeks) for children above the age of 12 months. WHO vaccination guidelines recommend administering the third dose of a diphtheria-containing vaccine at least 6 months after the second dose and a third dose of tetanus-containing vaccine at least 1 year after the second dose in order to mount durable protection [17–19]. While the DTCV survey coverage in MSs was similar for both children aged 1 to 6 years and those aged 7 to 14 years, the higher tetanus and diphtheria seroprotection in the 7- to 14-year-olds supports the hypothesis that this age group had previous access to routine immunizations prior to arrival in Bangladesh.

This study had certain limitations. Recall of campaign participation may be inaccurate. The study team attempted to minimize recall inaccuracies by using a calendar that incorporated vaccination campaigns alongside co-occurring migration (e.g., August and September mass in-migration), religious holidays (e.g., Eid), and cultural events (e.g., agricultural harvests). Findings from the study may not be generalizable to the larger Rohingya population because of the exclusion of 7- to 14-year-olds in Nayapara due to Ramadan as well as nonresponse—particularly in MSs among the 7- to 14-year-olds, who accounted for 89% of children not at home during the survey. The rubella and measles antibody MBA validation process is ongoing but nearly complete. The diphtheria antibody MBA is currently in the validation process and may not have direct correlation with protection, which is toxin neutralization measured by the Vero cell neutralization assay. Toxin neutralization can be due to antibodies other than immunoglobulin G (IgG); however, cross-reacting material (CRM)-conjugated vaccines were not used in vaccination campaigns, so interference from other vaccines is unlikely. Additionally, the tetanus MBA has already been validated, and diphtheria and tetanus results were similar [20]. Lastly, typically higher antibody levels for tetanus and diphtheria correlate with higher probability and duration of protection, but it is possible that an individual with low antibody levels is protected whereas an individual with high antibody levels could still acquire symptomatic disease [10]. The serologic results for any antigen provide a snapshot in time of immunity, and because antibody levels can wane over time, immunity gaps may be larger now than reported in this analysis.

WHO’s “Vaccination in Acute Humanitarian Emergences: a Framework for Decision-Making” provides guidance to governments and international partners on how to determine the risk of VPD outbreaks, which vaccines to deliver, and how to deliver them in the context of availability of human resources, cultural considerations, and appropriate social mobilization strategies [21]. In a rapidly evolving emergency situation, however, it can be difficult to operationalize these guidelines in a timely manner, as evidenced by the small proportion of children who received vaccination upon entry to Bangladesh and the remaining immunity gaps in the population, despite multiple vaccination campaigns. Multiple dose vaccinations are particularly difficult to deliver by campaigns alone, not only because of the intensity of resources necessary but because of campaign fatigue, a constant new cohort of children arriving who require multiple doses for full protection, and unique sociocultural issues. It is challenging to fully understand and adequately prepare for societal and cultural beliefs towards vaccination. Just prior to the survey, a rapid behavioral assessment, which included focus group discussions and key informant interviews, was conducted in 3 Rohingya camps. Findings from the assessment highlighted several major barriers to achieving high campaign coverage, including fear and misconception of becoming a Christian due to vaccination; safety concerns, including receipt of multiple vaccinations on the same day; and lack of sensitivity to cultural gender norms during vaccination procedures [8]. These findings, along with specific recommendations, were communicated to stakeholders and should continue to be considered as the Rohingya crisis evolves.

Children may still be at risk for future infections because of ongoing transmission of diphtheria and other infectious diseases in Rohingya camps and settlements. The guidelines on vaccination in acute humanitarian emergencies focus mainly on the use of mass vaccination campaigns, with instruction to resume or establish routine immunization whenever possible [21]. Although rapid scale-up and strengthening of existing routine immunization services is challenging because of political and resource constraints in Rohingya camps and settlements, reaching children under 2 years and delivering missed doses to older children to close these immunity gaps and prevent future VPD outbreaks is critical. Delivery of routine services is particularly important for closing gaps when serial doses are needed (e.g., 3 doses of DTCV). In emergency settings, vaccination upon entry, mass vaccination campaigns, and rapid scale-up of routine immunization services with strengthened social mobilization—including community leader involvement—is essential for protecting all children.

Based on the findings from this survey, despite multiple rounds of vaccination campaigns, VPD immunity gaps exist among children in both camps, particularly for diphtheria in MSs, which requires serial vaccinations to achieve maximum protection. In addition to scaling up routine immunization services, an additional tetanus-diphtheria campaign may be necessary in MSs to quickly address these remaining immunity gaps among children aged 1 to 14 years. Future campaigns and routine immunization services should be provided with respect to the sociocultural issues specific to the Rohingya population.

## Supporting information

S1 STROBE Checklist STROBE checklist for the vaccination coverage and seroprevalence study among forcibly displaced Rohingya children, Cox’s Bazar, Bangladesh, 2018. STROBE, Strengthening the Reporting of Observational Studies in Epidemiology.
(DOC)

S1 Table Vaccination campaign schedule. (DOCX)

S2 Table Reason for not getting vaccinated, among participants who did not get vaccinated during the third DTCV campaign, serologic and vaccination coverage survey among Rohingya children, Cox’s Bazar, Bangladesh, 2018. (DOCX)

S1 Appendix Sample size calculations and sampling methodology. (DOCX)

S1 Protocol Assessment of vaccination coverage and VPD serology and exposure to select parasitic diseases among forcibly displaced Rohingyas in Cox’s Bazar, Bangladesh, April–May 2018. (DOCX)

S1 Questionnaire Parent/infant caregiver vaccination questions, Cox’s Bazar, Bangladesh, April–May 2018. (DOCX)

S1 Data Vaccination coverage survey and seroprevalence dataset, Cox’s Bazar, Bangladesh, April–May 2018. (XLSX)

## Abbreviations

BCG: Bacillus Calmette-Guérin
CDC: Centers for Disease Control and Prevention
CI: confidence interval
CRM: cross-reacting material
DBS: dried blood spot
DTCV: diphtheria-tetanus containing vaccine
IgG: immunoglobulin G
MBA: multiplex bead assay
MR: measles-rubella
MS: makeshift settlement
OCV: oral cholera vaccine
PRNT: plaque reduction neutralization test
ROC: receiver operating characteristic
SRS: simple random sampling
STROBE: Strengthening the Reporting of Observational Studies in Epidemiology
UNHCR: United Nations High Commissioner for Refugees
VPD: vaccine-preventable disease
WHO: World Health Organization
