# Supplementary material for: Vaccination coverage survey and seroprevalence among forcibly displaced Rohingya children, Cox's Bazar, Bangladesh, 2018: A cross-sectional study
Source: PLoS Med. 2020 Mar 31;17(3):e1003071. doi: 10.1371/journal.pmed.1003071 (PMC7108726; doi:10.1371/journal.pmed.1003071)
Supplement: S1 STROBE Checklist — STROBE, Strengthening the Reporting of Observational Studies in Epidemiology. (DOC) [file pmed.1003071.s001.doc]

S1 STROBE Checklist

|  | Item No | Section, paragraph | Recommendation |
| --- | --- | --- | --- |
| **Title and abstract** | 1 | Abstract | (*a*) Indicate the study’s design with a commonly used term in the title or the abstract |
|  |  | Abstract | (*b*) Provide in the abstract an informative and balanced summary of what was done and what was found |
|  | | | Introduction |
| Background/rationale | 2 | Introduction, paragraphs 1-3 | Explain the scientific background and rationale for the investigation being reported |
| Objectives | 3 | Introduction, paragraph 3 | State specific objectives, including any prespecified hypotheses |
|  | | | Methods |
| Study design | 4 | Methods, paragraphs 1-3 | Present key elements of study design early in the paper |
| Setting | 5 | Methods, paragraphs 1-4 | Describe the setting, locations, and relevant dates, including periods of recruitment, exposure, follow-up, and data collection |
| Participants | 6 | NA | (*a*) *Cohort study*—Give the eligibility criteria, and the sources and methods of selection of participants. Describe methods of follow-up |
|  |  | NA | (*b*) *Cohort study*—For matched studies, give matching criteria and number of exposed and unexposed |
| Variables | 7 | Methods, paragraphs 6 | Clearly define all outcomes, exposures, predictors, potential confounders, and effect modifiers. Give diagnostic criteria, if applicable |
| Data sources/ measurement | 8 | Methods, paragraphs 6 | For each variable of interest, give sources of data and details of methods of assessment (measurement). Describe comparability of assessment methods if there is more than one group |
| Bias | 9 | Methods, paragraphs 6 | Describe any efforts to address potential sources of bias |
| Study size | 10 | S1 Appendix, paragraphs 1-2 | Explain how the study size was arrived at |
| Quantitative variables | 11 | Methods, paragraphs 6, S1 Appendix, paragraphs 5-6 | Explain how quantitative variables were handled in the analyses. If applicable, describe which groupings were chosen and why |
| Statistical methods | 12 | Methods, paragraphs 6 | (*a*) Describe all statistical methods, including those used to control for confounding |
|  |  |  | (*b*) Describe any methods used to examine subgroups and interactions |
|  |  |  | (*c*) Explain how missing data were addressed |
|  |  |  | (*d*) *Cohort study*—If applicable, explain how loss to follow-up was addressed |
|  |  |  | (*e*) Describe any sensitivity analyses |

| Section, paragraph | | | Results |
| --- | --- | --- | --- |
| Participants | 13 | Results, paragraphs 1 and 2 | (a) Report numbers of individuals at each stage of study—eg numbers potentially eligible, examined for eligibility, confirmed eligible, included in the study, completing follow-up, and analysed |
|  |  | Supplementary Table 1 | (b) Give reasons for non-participation at each stage |
|  |  | Supplementary Table 1 | (c) Consider use of a flow diagram |
| Descriptive data | 14 | Results, paragraphs 1 and 2 | (a) Give characteristics of study participants (eg demographic, clinical, social) and information on exposures and potential confounders |
|  |  |  | (b) Indicate number of participants with missing data for each variable of interest |
|  |  | NA | (c) *Cohort study*—Summarise follow-up time (eg, average and total amount) |
| Outcome data | 15 | NA | *Cohort study*—Report numbers of outcome events or summary measures over time |
|  |  | NA | *Case-control study—*Report numbers in each exposure category, or summary measures of exposure |
|  |  | Results, paragraphs 1 and 2 | *Cross-sectional study—*Report numbers of outcome events or summary measures |
| Main results | 16 | Results, paragraphs 3-10 | (*a*) Give unadjusted estimates and, if applicable, confounder-adjusted estimates and their precision (eg, 95% confidence interval). Make clear which confounders were adjusted for and why they were included |
|  |  | Results, paragraphs 3-10 | (*b*) Report category boundaries when continuous variables were categorized |
|  |  | NA | (*c*) If relevant, consider translating estimates of relative risk into absolute risk for a meaningful time period |
| Other analyses | 17 |  | Report other analyses done—eg analyses of subgroups and interactions, and sensitivity analyses |
|  | | | Discussion |
| Key results | 18 | Discussion, paragraph 1-4 | Summarise key results with reference to study objectives |
| Limitations | 19 | Discussion, paragraph 5 | Discuss limitations of the study, taking into account sources of potential bias or imprecision. Discuss both direction and magnitude of any potential bias |
| Interpretation | 20 | Conclusion, paragraph 2 | Give a cautious overall interpretation of results considering objectives, limitations, multiplicity of analyses, results from similar studies, and other relevant evidence |
| Generalisability | 21 | Conclusion, paragraphs 1-3 | Discuss the generalisability (external validity) of the study results |
|  | | | Other information |
| Funding | 22 | Funding | Give the source of funding and the role of the funders for the present study and, if applicable, for the original study on which the present article is based |
