# Supplementary material for: Vaccination coverage survey and seroprevalence among forcibly displaced Rohingya children, Cox's Bazar, Bangladesh, 2018: A cross-sectional study
Source: PLoS Med. 2020 Mar 31;17(3):e1003071. doi: 10.1371/journal.pmed.1003071 (PMC7108726; doi:10.1371/journal.pmed.1003071)
Supplement: S1 Table — (DOCX) [file pmed.1003071.s002.docx]

S1 Table. Vaccination campaign schedule

| Vaccination Campaign | Campaign Dates | Target Age (months (m) or years (y)) |
| --- | --- | --- |
| Measles-rubella | 16 Sept - 03 Oct 2017 | 6m - <15y |
| Bivalent oral polio | 16 Sept - 03 Oct 2017 | 0 - <5y |
| Oral cholera | 10 - 18 Oct 2017 | 1y - <5y |
| Oral cholera | 04 - 09 Nov 2017 | 1y - <5y |
| Bivalent oral polio | 04 - 09 Nov 2017 | 0 - <5y |
| Measles-rubella | 18 Nov - 05 Dec 2017 | 6m - <15y |
| Diphtheria-tetanus containing & bivalent oral polio | 12 - 31 Dec 2017 | 1.5m - <15y |
| Diphtheria-tetanus containing & bivalent oral polio | 27 Jan – 10 Feb 2018 | 1.5m - <15y |
| Diphtheria-tetanus containing & bivalent oral polio | 10 - 25 Mar 2018 | 1.5m - <15y |
| Oral cholera | 6 - 13 May 2018 | ≥1y |
