# Supplementary material for: Vaccination coverage survey and seroprevalence among forcibly displaced Rohingya children, Cox's Bazar, Bangladesh, 2018: A cross-sectional study
Source: PLoS Med. 2020 Mar 31;17(3):e1003071. doi: 10.1371/journal.pmed.1003071 (PMC7108726; doi:10.1371/journal.pmed.1003071)
Supplement: S2 Table — (DOCX) [file pmed.1003071.s003.docx]

S2 Table. Reason for not getting vaccinated, among participants who did not get vaccinated during the third diphtheria-tetanus containing vaccine (DTCV) campaign, serologic and vaccination coverage survey among Rohingya children, Cox’s Bazar, Bangladesh, 2018

| Reason child did not get vaccinated* | Makeshift Settlements | | Nayapara |
| --- | --- | --- | --- |
|  | **1-6 years**  **N=66** | **7-14 years**  **N=49** | **1-6 years N=16** |
| Caregiver was unaware of the vaccination campaign | 29 (44%) | 18 (37%) | 4 (25%) |
| Child was not available (e.g., not at home, sick) at time of vaccination | 27 (41%) | 13 (27%) | 5 (31%) |
| Child was afraid of needles/pain | 15 (23%) | 22 (45%) | 6 (38%) |
| Caregiver did not think vaccines were necessary | 5 (8%) | 4 (8%) | 0 (0%) |
| The child got fever/swelling after last vaccination | 3 (5%) | 0 (0%) | 1 (6%) |
| Caregiver was fearful of vaccines or of multiple injections | 2 (3%) | 3 (6%) | 0 (0%) |
| Caregiver thought that child received all required vaccines | 1 (2%) | 2 (4%) | 3 (19%) |
| Female caregiver or child not comfortable leaving the house | 1 (2%) | 2 (4%) | 0 (0%) |
| No one was available to take the child to get the vaccination | 1 (2%) | 1 (2%) | 0 (0%) |
| Caregiver or child was fearful about not going to heaven or being converted to Christianity | 0 (0%) | 1 (2%) | 0 (0%) |
| Female caregiver or child was not comfortable taking vaccine from a male vaccinator | 0 (0%) | 2 (4%) | 0 (0%) |
| Father or head of household did not allow | 0 (0%) | 0 (0%) | 0 (0%) |
| Vaccinator treated caregiver or child poorly | 0 (0%) | 0 (0%) | 0 (0%) |
| Other/Don’t know | 7 (11%) | 1 (2%) | 3 (19%) |

*Categories are not mutually exclusive
