# Supplementary material for: Vaccination coverage survey and seroprevalence among forcibly displaced Rohingya children, Cox's Bazar, Bangladesh, 2018: A cross-sectional study
Source: PLoS Med. 2020 Mar 31;17(3):e1003071. doi: 10.1371/journal.pmed.1003071 (PMC7108726; doi:10.1371/journal.pmed.1003071)
Supplement: S1 Appendix — (DOCX) [file pmed.1003071.s004.docx]

S1 Appendix: Sample size calculations and sampling methodology

*Nayapara sample size*

The following assumptions were made to estimate the expected number of children enrolled: 65% of households had at least one child aged 6 months-6 years (for DBS calculations, 60% had a child aged 1-6 years) and 65% of households had at least one child aged 7-14 years; (at the time of sample size calculations, the dates of the survey had not been determined and it was not known that data collection would occur during Ramadan). Household non-response rate (i.e., no one was home) was projected to be 20%, child non-response rate (e.g., refusal, child absent from home) was estimated to be 1% for all children for the survey component of the assessment, 10% for DBS specimen collection among children 1-6 years. Estimated coverage for three DTCV doses and the proportion of children with protective antibody levels for tetanus was 50%. Given these assumptions, the expected number of children enrolled for the coverage survey in Nayapara was 322 children aged 6 months-6 years, with 5.6% precision and 270 children aged 1–6 years for the serologic survey with 6.1% precision.

*Makeshift Settlements (MS) sample size*

The following assumptions were made to estimate the expected number of children enrolled: 65% of households had at least one child aged 6 months-6 years (for DBS calculations, 60% had a child aged 1-6 years) and 60% of households had at least one child aged 7-14 years. Household non-response rate (i.e., no one was home) was projected to be 5%, child non-response rate (e.g., refusal, child absent from home) was estimated to be 1% for all children for the survey component of the assessment, 10% for DBS specimen collection among children 1-6 years and 20% among children 7-14 years. Estimated coverage for three DTCV doses and the proportion of children with protective antibody levels for tetanus was 50%. Given these assumptions, the expected number of children enrolled for the survey was 445 children aged 6 months–6 years and 411 children aged 7-14 years in the settlements (Table 1). With a design effect of 1.7 (6 months-6 years) and 1.6 (7-14 years), this results in precision of 6.2% in both age groups. The enrollment for DBS was 374 children aged 1–6 years and 332 children aged 7-14 years, with design effects of 1.6 and 1.5, respectively and precision of 6.5% (1–6 years) and 6.7% (7-14 years). The expected total number of children enrolled in MS was 856 for the survey; among these children, 706 were for DBS specimen collection.

### *Household selection*

In MS, household lists per selected cluster were created in advance of data collection. On the day of data collection after it was confirmed that no households had left or joined the camp, 13 households were selected using a random number generator. With 55 clusters and 13 households per cluster, a sample of 715 households were selected, in slight excess of the ENA Software calculated sample of 706.

In Nayapara Refugee Camp, UNHCR randomly selected 411 registered households. An additional 113 unregistered households were randomly selected from enumerated lists of unregistered households residing within the camp, for a total sample of 524 households, with a ratio of registered to unregistered households proportional to the ratio observed in the population of the camp.

All households were eligible regardless of registration status, date of arrival, or presence of children. Survey teams attempted to survey 13 randomly selected households daily. To prevent data collectors who were observing Ramadan from over-exerting themselves, beginning May 19, 2018, the daily number of households surveyed was reduced to eight. Efforts were made to revisit absent households at least twice. Absent households were not replaced.

*Specimen testing: Multiplex bead assay*

Multiplex bead assay (MBA) procedures for coupling diphtheria toxoid, tetanus toxoid and measles virus N protein (MV-N) to beads have been described previously [1, 2]. Whole rubella virus was purchased from Meridian Life Sciences (Memphis, TN) and covalently coupled to beads in 2-*N*-Morpholinoethanesulfonic acid plus 0.85% NaCl at pH 5 using a ratio of 30 µg of whole virus per 12.5 X 10^6^ beads. To prepare specimens for testing, a 3 mm DBS was punched from each card using a Harris Uni-Core 3.00 mm Puncher and eluted in 125 µL Elution Buffer (1X PBS, 0.05% sodium azide, 0.3% Tween-20) overnight at 4°C in a polystyrene round bottom 96-well plate (Costar® 3799, Corning). A final serum of dilution of 1:400 was made in Buffer B (1X PBS, 0.5% polyvinyl alcohol, 0.8% polyvinylpyrrolidone, 0.5% casein, 0.3% Tween-20, 0.02% sodium azide) containing 3 µg/mL of *E. coli* lysate and incubated overnight. Assays were performed by incubating diluted specimens in duplicate in a 96-well plate with beads and then by detecting antigen-specific IgG with monoclonal anti-human IgG and IgG4 and streptavidin-linked R-phycoerythrin reporter as described previously [10, 20]. Antibody binding was detected as median fluorescence intensity (MFI) using a MAGPIX instrument. Background from a DBS blank was subtracted from the (MFI-BG). Specimens having a coefficient of variation of >15% between the MFI-BG of duplicate wells for any bead were repeated.

The WHO international standards obtained from the National Institute of Biological Standards and Control were used to create standard curves to convert MFI into International Units (IU) to assess protective levels of IgG for tetanus (TE-3), diphtheria (10/262), and rubella (67/182). Thresholds of protection are based on WHO-defined unit values [3-5]. For diphtheria, antibody concentrations of ≥0.01 IU/mL indicate the minimum level associated with protection against death from diphtheria and some protection against symptomatic disease, ≥0.1 IU/mL confers protection against symptomatic disease, and levels of >1.0 IU/mL are associated with long-term protection against diphtheria [6]**.** For tetanus, although there is no definitive immunological correlate of protection, ≥0.01 IU/mL antibody concentrations are usually considered protective, ≥0.1 IU/mL confers long-term protection against symptomatic disease and levels of >1.0 IU/mL are associated with life-long protection against tetanus [3]. For rubella, a cutoff level of ≥10 IU/mL is considered to provide long-term protection against disease. MV-N threshold of protection was defined using receiver operator characteristic curve of samples characterized by plaque reduction neutralization test (PRNT), (the current gold standard for measles protective immunity) run on the multiplex bead assay [7]. Seropositivity was defined as a PRNT result of 120 IU/mL or higher.

1. Scobie HM, Mao B, Buth S, Wannemuehler KA, Sorensen C, Kannarath C, et al. Tetanus Immunity among Women Aged 15 to 39 Years in Cambodia: a National Population-Based Serosurvey, 2012. Clin Vaccine Immunol. 2016;23(7):546-54. Epub 2016/04/08. doi: 10.1128/cvi.00052-16. PubMed PMID: 27053629; PubMed Central PMCID: PMCPMC4933773.

2. Ondigo BN, Muok EMO, Oguso JK, Njenga SM, Kanyi HM, Ndombi EM, et al. Impact of Mothers' Schistosomiasis Status During Gestation on Children's IgG Antibody Responses to Routine Vaccines 2 Years Later and Anti-Schistosome and Anti-Malarial Responses by Neonates in Western Kenya. Front Immunol. 2018;9:1402. Epub 2018/07/04. doi: 10.3389/fimmu.2018.01402. PubMed PMID: 29967622; PubMed Central PMCID: PMCPMC6015899.

3. World Health Organization. The Immunological Basis for Immunization Series, Module 3: Tetanus. Geneva: World Health Organization, 2018.

4. World Health Organization. The Immunological Basis For Immunization Series, Module 2: Diphtheria. Geneva: World Health Organization, 2009.

5. Skendzel LP. Rubella immunity. Defining the level of protective antibody. Am J Clin Pathol. 1996;106(2):170-4. Epub 1996/08/01. PubMed PMID: 8712168.

6. World Health Organization. Recommendations to assure the quality, safety and efficacy of diphtheria vaccines (absorbed). Geneva: World Health Organization, 2014.

7. Hummel KB, Erdman DD, Heath J, Bellini WJ. Baculovirus expression of the nucleoprotein gene of measles virus and utility of the recombinant protein in diagnostic enzyme immunoassays. J Clin Microbiol. 1992;30(11):2874-80. Epub 1992/11/01. PubMed PMID: 1452657; PubMed Central PMCID: PMCPMC270545.
