# Supplementary material for: Vaccination coverage survey and seroprevalence among forcibly displaced Rohingya children, Cox's Bazar, Bangladesh, 2018: A cross-sectional study
Source: PLoS Med. 2020 Mar 31;17(3):e1003071. doi: 10.1371/journal.pmed.1003071 (PMC7108726; doi:10.1371/journal.pmed.1003071)
Supplement: S1 Protocol — (DOCX) [file pmed.1003071.s005.docx]

**Assessment of vaccination coverage and vaccine-preventable disease serology and exposure to select parasitic diseases among forcibly displaced Rohingyas in Cox’s Bazar, Bangladesh April-May 2018**

**April-May 2018**

**Protocol**

**Contents**

[**1. Abstract/Summary** 2](#_Toc508232244)

[**2. Introduction** 4](#_Toc508232245)

[**3. Objectives** 7](#_Toc508232246)

[**4. Rationale** 8](#_Toc508232247)

[**5. Methodology** 10](#_Toc508232248)

[**6. Utilization of Results** 22](#_Toc508232249)

[**7. Facilities (Resources)** 22](#_Toc508232250)

[**8. Flow Chart (time frame)** 22](#_Toc508232251)

[**9. Ethical Implications** 23](#_Toc508232252)

[**10. References** 24](#_Toc508232253)

[**Annex D** 25](#_Toc508232254)

# **1. Abstract/Summary**

Provision of vaccines through immunization campaigns is crucial to control outbreaks of vaccine-preventable diseases (VPDs) because they provide individual protection and herd immunity. Several vaccination campaigns, targeting primarily children under 15 years in the forcibly displaced Rohingya camps and settlements, have been conducted to reduce the risk of disease and prevent outbreaks. However, outbreaks of measles and diphtheria have been documented in these communities. To better understand the impact of vaccination activities conducted in closing immunity gaps and preventing new outbreaks, the United Nations Children’s Fund (UNICEF), World Health Organization (WHO) and U.S. Centers for Disease Control and Prevention (CDC), in collaboration with the Bangladesh Government EPI Program and Institute of Epidemiology, Disease Control and Research (IEDCR), propose an assessment of vaccination coverage in a representative sample from the Rohingya camps and settlements using a vaccination coverage assessment questionnaire and collection of dried blood spot (DBS) for serologic analysis of immunity.

The vaccination coverage assessment questionnaire is an excellent tool to assess vaccination coverage and identify barriers for accessing immunization, but using it to estimate gaps in population immunity will be challenging in the Rohingya population because of the low retention of cards from vaccinations campaigns, difficulty recalling vaccine doses after multiple campaigns over numerous months, and the uncertainty of vaccination status in Myanmar. Thus, we propose to conduct a serological assessment by DBS simultaneously with the coverage assessment questionnaire in order to better estimate the proportion of individuals (especially among recent arrivals) that remain un- or under-protected against the diseases targeted by the vaccination efforts.

We propose to select a representative sample of households in Kutupalong Registered Camp, Nayapara Registered Camp, and the makeshift/informal settlements. In the formal camps, simple random sampling of households will take place; in the makeshift/informal settlements, we will use a multi-stage cluster approach. In each selected household, we will randomly select one child 6 months–6 years of age and one child aged 7 years–14 years. For each child, we will conduct a short interview in Rohingya in or near the home with the available caregiver using a standardized vaccine coverage assessment questionnaire to assess vaccines received during the campaigns and elsewhere, as well as barriers for receiving vaccination during the most recent vaccination campaign. Available vaccination cards will be reviewed to record documented vaccinations received. The expected number of children to be enrolled is ~1089 children aged 6 months–6 years and ~1055 children aged 7–14 years.

In a subset of children selected for the caregiver interview (expected number of children to be enrolled: ~914 children aged 1–6 years of age and ~787 children aged 7–14 years) we will collect 3–4 drops of blood using a single fingerprick. The drops of blood will be applied to filter paper for determination of antibodies to select VPDs. The dried blood spots contained in paper filters will be stored in Ziploc bags and plastic containers with desiccant bags to protect against humidity. The DBS samples will be sent to CDC laboratories in the United States. Specimens will be tested using a microneutralization assay for polio and a multiplex bead assay that allows determination of antibodies levels for several VPDs (measles, rubella, tetanus, and diphtheria) and parasitic diseases (malaria, lymphatic filariasis, trachoma, yaws) for testing on dried blood spots.

All participants will be asked for consent verbally in Rohingya using a standardized script and consent document. The survey objectives, time for participation (approximately 10 minutes for completing the questionnaire and 2–3 minutes for collection of DBS samples if selected), and risks/benefits of participation will be clearly explained to all the survey participants before enrolling them in the study. Minimal risk is a result of pain or small amount of bleeding at the DBS fingerprick site in children selected for DBS sample collection. No participants will be forced to provide information or DBS samples for the study; all participation will be voluntary. Personally identifying information will not be retained in the dataset; any remaining DBS sample will be destroyed after laboratory testing is completed.

The assessment of coverage and immunity will inform about the risk of additional VPD outbreaks in the camps and the threat to spread to the nearby Bangladesh host community. Data from this serosurvey can also inform about exposures to malaria, lymphatic filariasis, and yaws in camps and settlements, indicating the need for additional control programs. The results of this assessment will benefit the Rohingya population by:

1) Providing evidence about the remaining immunity gaps among the Rohingya population in order to identify VPD risks and target future vaccination efforts, both in the Rohingya population as well as the nearby host Bangladesh population;

2) Identifying barriers to vaccination in the Rohingya population, which, in turn, will help the microplanning of future immunization activities.

3) Provide information useful to malaria and parasitic control programs for future activities.

# **2. Introduction**

Since late August 2017, more than 650,000 forcibly displaced Rohingyas have moved into the Cox’s Bazar area in Bangladesh, joining an estimated 300,000 Rohingyas who had arrived during earlier waves of displacement [1]. The two pre-existing refugee camps, Kutupalong and Nayapara, and makeshift settlements have expanded with the new influx. New spontaneous settlements have also formed and are quickly growing. The dense concentration has put strain on infrastructure and services. Many partners—including the Bangladesh Ministry of Health and Family Welfare (MOH&FW), United Nations International Children’s Emergency Fund (Unicef) World Health Organization (WHO), United Nations High Commissioner for Refugees (UNHCR), International Organization for Migration (IOM), Médecins Sans Frontières (MSF), and others—are working to address the health issues facing this large and vulnerable population. The February 20, 2018 Mortality and Morbidity Weekly Bulletin on the health issues facing this displaced population in Cox’s Bazar in the first 7 weeks of 2018 reported large numbers of cases acute water diarrhea (36,533), acute respiratory infection (74,034), and suspected measles (713) [2].

Outbreaks of measles and diphtheria are ongoing. As of February 9, 2018, 5,560 suspected diphtheria cases and 38 deaths were reported [3]. Epidemiological data from the outbreak show that children under 15 years of age represent the highest proportion of cases at 73% [3]. Spillover of the diphtheria outbreak into the host community was reported in January 2018 [3].

In response to these events, the MOH&FW and partners managing the crisis have conducted the following activities in the camp and surrounding host communities:

- Expansion of treatment facilities to manage patients with diphtheria including administration of diphtheria antitoxin (DAT), and contact tracing for diphtheria cases;
- Implementation of Early Warning Alert and Response System (EWARS) for surveillance of diphtheria and other vaccine-preventable diseases (VPDs) at health posts within camps;
- Vaccination of new entries into Bangladesh from Myanmar with measles-rubella vaccine (MR) (6 months–14 years), and oral polio vaccine (OPV) (all ages); on Dec 23, 2017 the following antigens were added: Pentavalent vaccine (Penta) (6 weeks–6 years), pneumococcal conjugate vaccine (PCV) (6 weeks–6 years), and tetanus- diphtheria (Td) (7–14 years);
- Vaccination of close contacts of diphtheria cases at 19 fixed sites (January 2018);
- Planning for expansion of routine immunization service delivery to new health facilities in the Rohingya settlement areas;
- Additionally, beginning in September, 2017, the following vaccination campaigns have taken place:


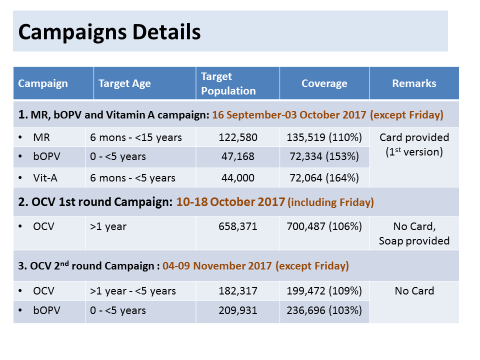


**
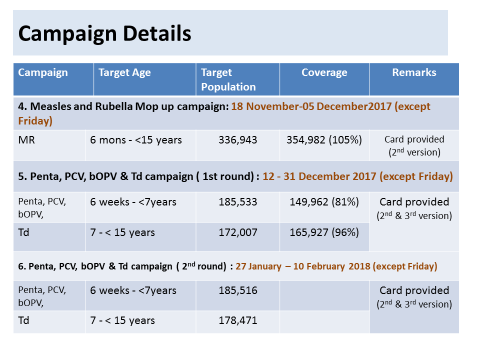
**

In addition, a third round of Penta & OPV (6 weeks–7 years) and Td (7–14 years) is planned for March 2018. Upon completion of the three rounds of Pentavalent, OPV and PCV vaccination, the MOH&FW and partners plan to establish routine immunization services and conduct additional immunization campaigns as necessary in order to reach and sustain levels of immunization coverage that protect the Rohingya children from VPDs and reduce the chance of outbreaks in the settlements and surrounding host community. Although a 2015 EPI Coverage Evaluation Survey reported that 80-84% of infants in Cox’s Bazar host community have received all of their scheduled vaccines, as of February 9, 2018 there have been 45 suspected diphtheria cases reported in the host community since November 2017 [3]. Bangladesh’s guidelines for routine immunization do not recommend a DTP booster after three doses of DTP during infancy. Therefore, there may be a large host population living in close proximity to the Rohingya settlements susceptible to VPDs as well.

In order to estimate the proportion of individuals (especially the recent arrivals) that remain un- or under-protected against the diseases targeted by the vaccination efforts, we propose to conduct an assessment of vaccination coverage and a serological assessment of immunity.

We also propose to use serological assays to measure active infection with malaria parasites and historical exposure to malaria and the neglected tropical diseases lymphatic filariasis (LF), trachoma, and yaws. The CDC multiplex assay for VPDs can include testing for these parasitic diseases without the need for additional blood collection. The Bangladesh-Myanmar border is one of the highest malaria transmission areas in South-East Asia. Estimating the burden of active infection and exposure to malaria in the Rohingya population will help guide whether additional prevention and control measures, such as bednet distribution and mass screen and treat are necessary. Bangladesh and Myanmar are both endemic for LF and are implementing activities to eliminate LF as recommended by WHO. Bangladesh has met criteria to stop mass drug administration (MDA), but Myanmar still has active transmission and has not yet stopped. It is important to conduct surveillance to minimize risk of re-introduction of LF to Bangladesh. If elevated levels of antibodies to LF are found in this population, we could provide recommendations for programmatic activities in the settlements to halt ongoing transmission. Myanmar has reported reaching trachoma elimination thresholds, but limited (if any) investigation has been undertaken in Rohingya populations; living conditions following their displacement across the border are unlikely to have lessened their risk for trachoma. If elevated levels of antibodies to *Chlamydia trachomatis*, the causative agent of trachoma, are found in children in this population, we could provide recommendations for further trachoma investigations or MDA in the settlements. Although only 5,000 cases of yaws are reported each year in South-East Asia [4], the prevalence of yaws in this difficult-to-reach and underserved population may vary. Characterizing yaws transmission in the settlements is crucial for providing treatment guidelines.

# **3. Objectives**

**General objectives**

- Assess whether vaccine campaigns have had the expected impact on population immunity among children 6 months–14 years of age through:
  - Childhood vaccination coverage assessment
  - Serological assessment of immunity for several VPDs
- Identify barriers to accessing and utilizing vaccination services in the camps
- Assess exposure to malaria and other parasitic and neglected tropical diseases

**Specific objectives**

**Primary**

- - Determine the proportion of Rohingya children aged 6 months–6 years who received 0, 1, 2, or 3 doses of pentavalent vaccine and 0, 1, 2, or 3 doses of OPV in campaigns since August 2017
  - Determine the proportion of Rohingya children aged 1 year–6 years with protective antibody titers for poliovirus, tetanus, diphtheria, measles, and rubella
  - Determine the proportion of Rohingya children aged 7 years–14 years who received 0, 1, 2, or 3 doses of Td vaccine in campaigns since August 2017
  - Determine the proportion of Rohingya children aged 7 years–14 years with protective antibody titers for poliovirus, tetanus, diphtheria, measles, and rubella

**Secondary**

- - Determine the proportion of Rohingya children aged 6 months–14 years who received measles/rubella, and OCV doses in campaigns since August 2017
  - Determine the proportion of Rohingya children aged 7 years–14 years who received 0, 1, 2, or 3 doses of OPV in campaigns since August 2017
  - Identify reasons for not being vaccinated in the last pentavalent/Td vaccination campaign among Rohingya children aged 6 months–14 years
  - Determine the proportion of Rohingya children aged 6 months–14 years who received vaccination upon entry into Bangladesh
  - Determine the proportion of Rohingya children aged 6 months–14 years who received vaccination in Myanmar
  - Estimate malaria parasite prevalence in children aged 1 year–14 years
  - Estimate prevalence of exposure to neglected tropical diseases (e.g., lymphatic filariasis, trachoma, and yaws) in children aged 1 year–14 years

# **4. Rationale**

Provision of vaccines through immunization campaigns is crucial to control outbreaks of vaccine-preventable diseases (VPDs) because they provide individual protection and herd immunity. Several vaccination campaigns, targeting primarily children under 15 years in the forcibly displaced Rohingya camps and settlements, have been conducted to reduce the risk of disease and prevent outbreaks. However, outbreaks of measles and diphtheria have been documented in these communities. To better understand the impact of vaccination activities conducted in closing immunity gaps and preventing new outbreaks, the United Nations Children’s Fund (UNICEF), World Health Organization (WHO) and U.S. Centers for Disease Control and Prevention (CDC), in collaboration with the Bangladesh Government EPI Program and Institute of Epidemiology, Disease Control and Research (IEDCR), propose an assessment of vaccination coverage in a representative sample from the Rohingya camps and settlements using a vaccination coverage assessment questionnaire and collection of dried blood spot (DBS) for serologic analysis of immunity.

The vaccination coverage assessment questionnaire is an excellent tool to assess vaccination coverage and identify barriers for accessing immunization, but using it to estimate gaps in population immunity will be challenging in the Rohingya population because of the low retention of cards from vaccinations campaigns, difficulty recalling vaccine doses after multiple campaigns over numerous months, and the uncertainty of vaccination status in Myanmar. Thus, we propose to conduct a serological assessment by DBS simultaneously with the coverage assessment questionnaire in order to better estimate the proportion of individuals (especially among recent arrivals) that remain un- or under-protected against the diseases targeted by vaccination efforts.

Recent laboratory advances allow testing for serum antibodies for polio, other VPDs, and parasitic diseases in DBS. DBS can be collected on filter paper by fingerpricks, which require minimum amount of blood sampled and are easier to obtain from children. In addition, antibodies remain stable once dried on filter paper, and DBS are easier to process and transport in low-resource settings because they do not require centrifugation or cold chain.

The assessment of coverage and immunity will inform about the risk of additional VPD outbreaks in the camps and the threat to spread to the nearby Bangladesh host community. Data from this serosurvey can also inform about exposures to malaria, lymphatic filariasis, and yaws in camps and settlements, indicating the need for additional control programs. The results of this assessment will benefit the Rohingya population by:

1) Providing evidence about the remaining immunity gaps among the Rohingya population in order to identify VPD risks and target future vaccination efforts, both in the Rohingya population as well as the nearby host Bangladesh population;

2) Identifying barriers to vaccination in the Rohingya population, which, in turn, will help the microplanning of future immunization activities.

3) Provide information useful to malaria and parasitic control programs for future activities.

# **5. Methodology**

**Overview**

We propose to select a representative sample of households in Kutupalong Registered Camp, Nayapara Registered Camp, and the makeshift/informal settlements. In the formal camps, simple random sampling of households will take place; in the makeshift/informal settlements, we will use a multi-stage cluster approach. In each selected household, we will randomly select one child 6 months–6 years of age and one child aged 7–14 years. For each child, we will conduct a short interview in Rohingya in or near the home with the available caregiver using a standardized vaccine coverage assessment questionnaire to assess vaccines received during the campaigns and elsewhere, as well as barriers for receiving vaccination during the most recent vaccination campaign. Available vaccination cards will be reviewed to record documented vaccinations received. The expected number of children to be enrolled is ~1089 children aged 6 months–6 years and ~1055 children aged 7–14 years.

In a subset of children selected for the caregiver interview (expected number of children to be enrolled: ~914 children aged 1–6 years of age and ~787 children aged 7–14 years), we will collect 3–4 drops of blood using a single fingerprick. The drops of blood will be applied to filter paper for determination of antibodies to select VPDs. The dried blood spots contained in paper filters will be stored in Ziploc bags and plastic containers with desiccant bags to protect against humidity. The DBS samples will be sent to CDC laboratories in the United States. The specimens will be tested using a microneutralization assay (polio) and a multiplex bead assay that allows determination of antibodies levels for several VPDs (measles, rubella, tetanus, and diphtheria) and parasitic and neglected tropical diseases (malaria, lymphatic filariasis, trachoma, yaws) for testing on dried blood spots.

**Study Design**

For purposes of this vaccination assessment, the camps will be divided into the following: Kutupalong Registered Camps, Nayapara Registered Camps, and the informal/makeshift settlements. Surveys within the registered refugee camps (Kutupalong and Nayapara) will apply simple random sampling of households. The survey in the informal/makeshift settlements will use a three-stage cluster sampling design. Children aged 6 months–14 years will be eligible to participate. There are no exclusion criteria.

1. **Kutupalong and Nayapara Refugee Camps (Simple Random Sampling)**
2. **Household Selection**

The most up-to-date lists of registered refugees/households will be used to randomly select households 625 housedholds in both Kutupalong and Nayapara Refugee camps.

**b. Child selection**

During data collection, team members will generate a line list of children in the household aged 6 months–6 years and 7 years–14 years, ordered youngest to oldest. The team will select one child randomly (using simple random sampling) from each age group using a random number generator. Children will be assigned a unique study ID. The caregiver of the selected child(ren) will be consented, enrolled, and interviewed (if consent obtained) using the vaccination coverage assessment questionnaire. DBS samples will also be collected on selected children aged 1y–14y.

1. **Sample size:**

Logistics and available resources determined that the maximum number of households that can be visited by the field teams is 625 households in both Kutupalong and Nayapara camps. To estimate the expected number of children enrolled we made the following assumptions (Table 1):

- 65% of households will have at least one child aged 6m–6y (for DBS calculations, 60% will have a child aged 1y–6y) and 65% of households will have at least one child aged 7y–14y
- Household non-response rate (i.e., no one home) is 20%
- Child non-response rate (e.g., refusal, child absent from home) is 1% for all children for the survey component of the assessment (primary respondent for the questionnaire is the mother or caregiver); for DBS specimen collection, non-response is estimated at 10% for children 1y–6y and 30% for children aged 7y–14y
- Coverage for 3 pentavalent doses and the proportion of children with protective antibody levels for tetanus is 50%

Given these assumptions, the expected number of children enrolled for the survey is 322 children aged 6m–6y and 322 children aged 7y–14y in each of the camps (Table 1). This results in a 95% Wilson-Score confidence with precision of 5.6% in each age group. The expected number of enrolled children for DBS is 270 children aged 1y–6y and 228 children aged 7y–14y in both the Kutapalong and Nayapara camps (Table 1); precision is 6.1% and 6.7% in each age group, respectively.

The expected total number of children enrolled in both Kutupalong and Nayapara camps is 1,288 for the survey; among these children, 996 will be enrolled for DBS specimen collection.

Table 1. Kutupalong and Nayapara sample size calculations

| Component | Age group | # Households Visited | Proportion of households with an eligible child | Household Non-response (i.e., no one home) | Child Non-response (i.e., refusal, not home) | # children expected to be enrolled | Coverage/ seroprevalence | Precision |
| --- | --- | --- | --- | --- | --- | --- | --- | --- |
| Coverage survey | 6m–6y | 625 | 65% | 20% | 1% | 321.8 | 50% | 5.6% |
| Coverage survey | 7y–14y | 625 | 65% | 20% | 1% | 321.8 | 50% | 5.6% |
| DBS | 1y–6y | 625 | 60% | 20% | 10% | 270.0 | 50% | 6.1% |
| DBS | 7y–14y | 625 | 65% | 20% | 30% | 227.5 | 50% | 6.7% |

1. **Makeshift and Informal Settlements (Three-Stage Cluster Sampling)**
2. **First Stage Sampling – Selection of Clusters**

Population estimates and maps with blocks/subdivisions for the makeshift/informal settlements for Rohingya will be obtained from the intersectoral coordination group (ISCG). Rohingya persons living among host (Bangladeshi) communities will not be included.

Logistics and available resources determined that the maximum number of clusters of 13 households is 56. Clusters will be defined using zones/blocks and other subdivisions existing in recent maps. Fifty-six (56) clusters will be selected using population proportional to estimated size (PPES) with ENA software.

Reserve clusters may be implemented only if ≥10% of clusters cannot be reached or if ≥20% of the sample size of children is not reached due to higher than expected non-response or refusal.

1. **Second Stage Sampling – Household Selection**

One member of the survey team will arrive in each of the clusters one day before teams are scheduled to interview the cluster. Blocks/zones larger than 200 households will be divided into smaller segments. This division can be done based on existing administrative units (neighborhoods, etc.), natural landmarks (river, road, mountains, etc.), or public places (market, schools, churches, mosques, temples, etc.). One segment will be chosen at random, applying PPS.

On arrival, the team member will identify the local community leader (Mahji) for the block and determine if there is an existing list of households for the selected block or segment of block. If a household list is available, the team member will update the list (adding any households that have joined recently and removing households that have left). If a household list is not available, the team member will work with the community leader to create the list.

Once the list is updated/created, the team member will use a random number generator to select 13 households from the list. A community leader will be selected to help escort the survey team to the selected households the following day.

1. **Third stage sampling – Child selection**

During data collection, team members will generate a line list of children in the household aged 6m–6y and 7y–14y, ordered youngest to oldest. The team will select one child randomly (using simple random sampling) from each age group using a random number generator. Children will be assigned a unique study ID. The caregiver of the selected child(ren) will be consented, enrolled, and interviewed (if consent obtained) using the vaccination coverage assessment questionnaire. DBS samples will also be collected on selected children aged 1y–14y.

1. **Sample size for makeshift camps:**

Thirteen (13) households in 56 clusters will result in a total of 728 households visited. To estimate the expected number of children enrolled we made the following assumptions (Table 1):

- 65% of households will have at least one child aged 6m–6y (for DBS calculations, 60% will have a child aged 1y–6y) and 60% of households will have at least one child aged 7y–14y
- Household non-response rate (i.e., no one home) is 5%
- Child non-response rate (e.g., refusal, child absent from home) is 1% for all children for the survey component of the assessment (primary respondent for the questionnaire is the mother or caregiver); for DBS specimen collection, non-response is estimated at 10% for children 1y–6y and 20% for children aged 7y–14y
- Coverage for 3 pentavalent doses and the proportion of children with protective antibody levels for tetanus is 50%

Given these assumptions, the expected number of children enrolled for the survey is 445 children aged 6m–6y and 411 children aged 7y–14y in the settlements (Table 1). With a design effect of 1.7 (6m–6y) and 1.6 (7y–14y), this results in a 95% Wilson-Score confidence with precision of 6.2% in both age groups. The enrolment for DBS is 374 children aged 1y–6y and 332 children aged 7y–14y in the settlements (Table 1); the design effect is 1.6 and 1.5, respectively, resulting in a precision of 6.5% (1y–6y) and 6.7% (7y–14y).

The expected total number of children enrolled in the makeshift settlements is 856 for the survey; among these children, 706 will be enrolled for DBS specimen collection.

Table 3. Makeshift camp sample sizes:

| Component | Age group | # Clusters | # Housholds per cluster | Proportion of households with an eligible child | Household Non-response (i.e., no one home) | Child Non-response (i.e., refusal, not home) | # children expected to be enrolled | Design effect** | Precision |
| --- | --- | --- | --- | --- | --- | --- | --- | --- | --- |
| Coverage survey | 6m–6y | 56 | 13 | 65% | 5% | 1% | 445.0 | 1.7 | 6.2% |
| Coverage survey | 7y–14y | 56 | 13 | 60% | 5% | 1% | 410.8 | 1.6 | 6.2% |
| DBS | 1y–6y | 56 | 13 | 60% | 5% | 10% | 373.5 | 1.6 | 6.5% |
| DBS | 7y–14y | 56 | 13 | 60% | 5% | 20% | 332.0 | 1.5 | 6.7% |

**Survey Teams and Supervision**

The survey will be implemented by six teams, each consisting of 1 team leader, 1 interviewer, 1 person to conduct DBS, and 1 advance (for mapping and listing). Additional persons will be trained and kept as reserve team members. Co-investigators and staff from MOHF&W, Unicef, WHO, and CDC will serve as supervisors.

Incentives will be paid to a community member on each survey day for support in identification of households, mapping of zone boundaries, and support in carrying of equipment.

**Survey Team Training**

All team members, supervisors, and reserve staff will participate in the training. The survey team will receive a 5 days training (1–5 April 2018) which includes classroom training, standardization test, and field test. In the training, the field enumerators will be trained on survey objectives, household selection techniques, demonstration of dried blood spot technique, content of the questionnaire, and use of mobile data collection. The training will include both lecture and practical sessions.

A field test will be conducted a day before the actual survey in extention areas of Kutupalong Makeshift Settlement. The questionnaire will be translated into Bengali and administered in the local Rohingya language (as there is no written script for the Rohingya language).

**Data Collection, Management and Analysis Plan**

Data collection will take place in Kutupalong refugee camp over the course of about 1 week, in makeshift and informal settlements over the course of 3–4 weeks, and Nayapara refugee camp over the course of about 1 week.

Data will be collected on tablets (Lenovo). Data will be uploaded daily to a Kobo server to enable remote monitoring of data quality. All teams will carry hard copies of the questionnaires as back-ups in case the tablet fails at any point.

A daily random check of entered data will be conducted by the survey manager. Team leaders with respective program personnel will ensure quality data collection at field level.

Vaccination coverage, seroprevalence and respective 95% confidence intervals will be estimated for 6m–6yr old children and 7–14yr old children using survey methods that account for the cluster design and sampling weights.

**Dried Blood Spots (DBS)/Serologic Assessment**

1. **Collection of dried blood spot (DBS) specimens**

The team will assign the HH and the children the appropriate ID numbers. The lab technician collecting DBS will ensure that children selected for DBS has been appropriately consented, enrolled, and a unique identifier (ID) assigned (described elsewhere).

The lab technician and assistant will place a clean mat on a table or rug, and organize the required supplies on the mat, including a lancet, alcohol swabs, sterile gauze, band-aid, DBS card with two specimen labels pre-attached, labels for the unique ID, and marker. The lab technician will attach on the DBS card two labels with the child’s unique ID number. The unique ID should be recorded alongside the child’s questionnaire in the data collection tablet.

The technician will clean his/her hands with sanitizer, and don new gloves. After cleaning a fingertip from the child’s second or third finger with an alcohol swab, the technician will do a finger prick using a single-use blood lancet. Holding the finger downside above one circle on the DBS card and applying gentle pressure without squeezing, the technician will ensure that one drop of free-flowing blood falls onto and saturates one of the 15 millimetre circles on the DBS card. The process will be repeated to complete at least three of the five circles (one drop per circle).

Once the dried blood spots have been filled, the technician or assistant, without touching the blood spots, will place the DBS card in a portable drying rack inside a plastic container to allow drying. Once this is completed, the technician will apply a sterile gauze to the prick site until bleeding stops.

If there is another eligible child in the household, the technician will conduct another prick as explained above and store the DBS card in the drying rack as before.

1. **Specimen Transport and storage**

The specimens collected during one day will be kept in drying racks inside plastic boxes to allow them to dry without exposure to dust, insects, or humidity. At the end of the day, the cards will be transported to a health facility or coordination site and will be allowed to dry for at least 4 hours in the racks. The cards will be cut into two portions, ensuring one circle in one portion and 2 circles in the other portion (do not cut circles in half). Each card portion should have a unique ID label and a specimen label. The card portions will be placed in independent glassine bags and then stored in a zip-lock bag containing a sachet with 5g of silica gel desiccant (Uline Inc Silica gelpacket, Aldrich Chemical) and a humidity indicator card.

The single specimen bags will be placed in large Ziploc bags with additional desiccant packs to protect from humidity. A study coordinator will check the humidity indicators weekly, and change desiccant packs when needed.

At the end of the sample collection the DBS cards will be shipped to CDC laboratories for testing.

1. **Specimen testing**
2. **Determination of Poliovirus antibodies**

At the CDC laboratory in Atlanta, determination of neutralizing antibody titers against poliovirus types 1, 2 and 3 in oral fluids and serum will be done using standard micro-neutralization assays adapted for dried blood spots [5]. Briefly, three 6-mm punches will be obtained from a circle in the DBS card, and eluted with Eagles’s Minimum Essential Medium and Fetal bovine Serum, for serum extraction. The serum will be aliquoted into 384-well plates that contain certain amounts of wild poliovirus of each of the three serotypes. The principle of the test is that the anti-poliovirus antibodies in a serum sample will bind to the virus and block infection of susceptible cells. Because poliovirus is cytopathic, virus that is not bound by antibody infects and lyses cells. The amount of neutralizing antibody is quantitated as a titer based on the last serum dilution to protect susceptible cell culture wells from poliovirus infection and cytopathic effect. Each test serum is run in triplicate and diluted from 1:8 to 1:1024.

The test takes approximately 7 days to complete from the elution of dried blood spot punches to adding luminescent reagent and reading plates for cytopathic effect. In each run there is a control serum which is pooled from serum samples with high neutralizing antibody titers to each Sabin poliovirus. Due to the size of the wells and accuracy required for liquid handling on the scale that is necessary for 384-well plates, this procedure cannot be performed manually.

A reciprocal titer of ≥8 indicates the presence of neutralizing antibodies, and is considered protective against poliomyelitis.

1. **Determination of antibodies against other vaccine preventable and parasitic diseases by Multiple Bead Assay (MBA)**

Luminex analysis or Multiple Bead Assay

Luminex platforms have been used for years to measure levels of multiple analytes in biological samples. Fluorescent microspheres can be readily conjugated with protein ligands, and the different spectral signatures of the labeled beads permit to include up to 100 distinct antigens in a single assay well for quantitative determinations of bound antibody. Luminex-based antibody assays are generally more sensitive than conventional ELISAs, have a wider dynamic range and are highly reproducible from assay to assay. The CDC laboratory has been developing assays for the determination of antibodies (IgG) against several vaccine preventable, parasitic, and neglected tropical diseases.

The assay in the Luminex testing includes antigens to measure protective antibody levels for tetanus, diphtheria, measles and rubella. Tetanus, diphtheria and rubella thresholds of protection are based on WHO unit value cutoffs and standards [6]. The measles virus nucleoprotein (MV-N) is an important immune target during measles infection and has shown good potential for use in diagnostics in lieu of whole virus [7].

Antigens to be included for malaria IgG serology testing will include both short and long-term markers for exposure. For lymphatic filariasis three antigens will be used: Wb123, Bm14 and Bm33. Wb123 is specific for *Wuchereria bancrofti* the main causative agents of LF; it is highly expressed in the larval stage transmitted by mosquitos, and could be a marker of ongoing transmission. Bm14 and Bm33 are sensitive markers of any historical exposure [9].

For trachoma, two antigens derived from the causative agent *Chlamydia trachomatis* are validated for use on the MBA, pgp3 and CT694. pCT03 (pgp3) is one of the eight total ORFs on the highly-conserved cryptic plasmid which is rarely found in *Chlamydia pneumonia* isolates. CT694 is a secreted protein and has been found to be involved in pathogenesis. CT694 manipulates host proteins by acting as a T3S-dependent substrate, but its exact function is not known. At the individual level high antibody levels indicate active or previous infection. At the community level, communities with higher trachoma prevalence have higher pgp3 and CT694 antibody responses. Lack of antibody in young children in a community may be indicative of interruption of transmission by MDAs and the SAFE strategy [10].

For yaws, two antigens will be assessed, rp17 which is a sensitive marker of historical infection and TmpA which is a marker or recent or ongoing infection. Yaws is an infectious disease caused by *Treponema pallidum* ssp*. pertenue*, which mainly affects children in rural communities in the humid tropics. WHO has targeted yaws eradication by 2020 and has developed the Morges strategy, which is comprised of mapping the disease at a community level and subsequently treating the entire population of communities where yaws is endemic. The program goals are to interrupt transmission in all countries where the disease is currently endemic and to certify that all previously countries where the disease was endemic are yaws free. Detection of antibodies against rp17 and TmpA integrated into serosurveillance for other diseases can be used to provide evidence for lack of transmission of yaws following these interventions [11].

Methods for DBS elution

A 6-mm punch will be extracted from each circle on the DBS card to obtain approximately 10 µL of dried whole blood. As previously described [8], the filter paper will be eluted overnight in buffer consisting of phosphate-buffered saline (PBS) with Tween 20, polyvinyl alcohol, polyvinylpyrrolidone, and lysate from *Escherichia coli*.

Multiplex bead assay

After overnight storage at 4°C, the final dilutions, which will represent a serum dilution of approximately 1:400, will be incubated with antigen-coupled beads in a 96-well formatted plate.

Antigen-specific IgG is detected on the coupled beads using biotin-linked monoclonal anti-human IgG and IgG4 antibodies. IgG is quantitatively reported through incubation with streptavidin-linked R-phycoerythrin.

Using a reader compatible with Luminex reagents (e.g., MAGPIX), the median fluorescence intensity (MFI) from duplicate wells will be recorded. Background from a DBS blank is subtracted from the median fluorescent intensity. Positive- and negative-control serum dilutions are also included on each plate to monitor assay performance.

WHO international standards obtained from the National Institute of Biological Standards and Control (NIBSC) will be used to create standard curves to identify MFI cutoffs representing protective levels of IgG for tetanus (TE-3), diphtheria (10/262), and rubella (67/182). Thresholds of protection are based on WHO-defined unit values [12-14]. Measles (MV-N) threshold of protection was defined using ROC curve of samples characterized by plaque reduction neutralization test (the current gold standard for measles protective immunity) run on the multiplex bead assay [CDC unpublished]. Cutoffs for malaria, trachoma, yaws and lymphatic filariasis antigens can be determined using known seronegative specimens from different sources.

Below is a table showing the sensitivity and specificity of the determination of antibodies by MBA against the gold standard, when available.

| **Disease** | **Pathogen** | **Antigen** | **Gold standard** | | **Specificity** | |
| --- | --- | --- | --- | --- | --- | --- |
|  | | | **Positive panel Classification** | **Sensitivity (95% CI)** | **Negative panel Classification** | **Specificity (95% CI)** |
| Trachoma | Chlamydia trachomatis | Pgp3 | Amplicor PCR (active infection) | 91%  (62% - 98%) | Non-endemic population | 98%  (93% - 99%) |
|  |  | Ct694 | Amplicor PCR (active infection) | 91%  (62% - 98%) | Non-endemic population | 98%  (93% - 99%) |
| Yaws | Treponema palladium | r-p17 | TPP(H)A+, RPR+ (active infection) | 100%  (97%-100%) | Non-endemic population | 100%  (98%-100%) |
|  |  | TmPA | TPP(H)A+, RPR+ (active infection) | 97%  (92%-99%) | Non-endemic population | 100%  (98%-100%) |
| Lymphatic Filariasis | Wuchereria bancrofti | Wb123 | blood smear microfilaria + (active infection) | 82%  (71%-90%) | Non-endemic population | 100%  (96%-100%) |
|  |  | Bm14 | blood smear microfilaria + (active infection) | 95%  (87%-98%) | Non-endemic population | 93%  (86%-98%) |
|  |  | Bm33 | blood smear microfilaria + (active infection) | 94%  (85%-97%) | Non-endemic population | 98%  (92%-99%) |
| Malaria | Plasmodium falciparum | Pf MSP1-19 | blood smear + (active infection) | 75%  (59%-87%) | Non-endemic population | 100%  (96%-100%) |
|  |  | Pf CSP | no data | no data | no data | no data |
|  |  | Pf LSA1 | no data | no data | no data | no data |
|  |  | Pf AMA1 | no data | no data | no data | no data |
|  | Plasmodium ovale | Po MSP1-42 | no data | no data | no data | no data |
|  | Plasmodium vivax | Pv MSP1-19 | blood smear + (active infection) | 94%  (81%-98%) | Non-endemic population | 99%  (94%-100%) |
| Tetanus | Clostridium tetani | tetanus toxoid | Double antigen ELISA (SSI, Denmark)* | 99% | Not available | 92% |
| Measles | Measles virus | MV-N | Vero cell toxin neutralization assay (DRL, UK)* | 84% | Vero cell toxin neutralization assay (DRL, UK)* | 95% |
| Rubella | Rubella virus | whole virus | Plaque reduction neutralization test (PRNT) | 88% | Plaque reduction neutralization test (PRNT) | 80% |
| Diphtheria | Corynebacterium diphtheriae | diphtheria toxoid | Enzygnost anti-rubella IgG ELISA (Siemens, Germany) | 97% | Double antigen ELISA (SSI, Denmark)* | 94% |

1. **Multiplexed photo-induced electron transfer (PET)-PCR for diagnosis of malaria infection**

Nucleic acid amplification tests (molecular tests), such as PCR-based assays, have been shown to have superior sensitivity and specificity compared to microscopy and rapid diagnostic tests, as they can detect very low levels of malaria parasitaemia. However, the cost and complexity of molecular tests has impeded its worldwide use for epidemiological studies and surveillance studies. Recent PCR assays such as the multiplexed photo-induced electron transfer (PET)-PCR, are less complex and have shown to be as robust and cost-effective compared to the nested PCR [15]. This test will be applied to the DBS collected in this study as a confirmatory test for malaria infection status.

First the DNA will be eluted from dried blood with a dilution buffer. Then the DNA will be extracted from the sample using the QIAamp DNA Mini Kits (QIAGEN, Valencia, CA, USA) as described by the manufacturer.

The samples will be screened using the multiplex PETPCR assay as described by Luchi et a. [16]. Briefly, the amplification of Plasmodium genus (forward and reverse primers and 80 nM of the species specific probes will be prepared. Then the following cycling conditions will be applied: an initial step at 50°C for 2 min, 95C° for 10 min, and 45 cycles of 95°C for 15 sec, and 60°C for 1 min. All assays were performed using the Applied Biosystems 7500 thermocyclers (Life Technologies, Grand Island, NY, USA).

A cut-off CT value of 40 will be used to indicate a positive result, i.e. active malaria infection. These findings will be compared with the IgG and antigen detection serological tests.

# **6. Utilization of Results**

The assessment of coverage and immunity will inform about the risk of additional VPD outbreaks in the camps and the threat to spread to the nearby Bangladesh host community. The results of this assessment will benefit the Rohingya population by:

1) Providing evidence about the remaining immunity gaps among the Rohingya population in order to identify VPD risks and target future vaccination efforts, both in the Rohingya population as well as the nearby host Bangladesh population;

2) Identifying barriers to vaccination in the Rohingya population, which, in turn, will help the microplanning of future immunization activities.

3) Provide information useful to malaria and parasitic control programs for future activities.

# **7. Facilities (Resources)**

All study materials (e.g., backpacks and pens; paper questionnaires; tablets; materials for household line listing, household and child selection; wet weather gear; and, DBS sample collection (e.g., gloves, hand sanitizer, gloves, alcohol swabs, single use lancets, DBS filter paper, sample storage materials) will be provided by the study organizers. Laboratory materials (e.g., reagents, disposable laboratory supplies, laboratory testing equipment, etc.) will be provided by the CDC.

# **8. Flow Chart (time frame)**

The timeframe and activities anticipated are outlined below:

| **Month and Year** | **Activity** |
| --- | --- |
| February–March 2018 | Develop protocol; submit protocol to ethical review |
| March 2018 | Hire study team personnel; procure study materials and supplies |
| March 2018 | Develop training materials for data and sample collection |
| April 2018 | Train study team |
| April–May 2018 | Conduct data and sample collection |
| May 2018 | Ship samples to CDC |
| May–June 2018 | Conduct data analysis; disseminate preliminary findings |
| May–August 2018 | Test laboratory samples at CDC; finalize data analysis; disseminate final report |

# **9. Ethical Implications**

The proposed assessment is minimal risk and does not present significant concerns regarding ethical treatment of study participants. The proposed assessment has been determined to be non-research by the CDC Center for Global Health Office.

All participants will be asked for consent verbally in Rohingya using a standardized script and consent document (see Annex D). The survey objectives, time for participation (per child, approximately 10 minutes for completing the questionnaire and 2–3 minutes for collection of DBS samples if selected), and risks/benefits of participation will be clearly explained to all the survey participants before enrolling them. Minimal risk is a result of pain or small amount of bleeding at the DBS fingerprick site in children selected for DBS sample collection. No participants will be forced to provide information or DBS samples for the study; all participation will be voluntary.

Personally identifying information will not be retained in the dataset. Any remaining DBS samples will be destroyed following completion of the laboratory testing. The enumerators will be highly committed to the respondents to keep the privacy of survey participants’ information and sources of data as well as will make their heartiest endeavor to be unbiased in collecting data.

# **10. Analysis plan**

| **Household vaccine coverage survey** | | | |
| --- | --- | --- | --- |
| **#** | **Result** | **Analysis** | **Details** |
| 1 | Denominators | n/% visited, enrolled, refused, not present: flowchart | Overall, by camp, day, team, age group |
| 2 | Demographics | n/% gender, median/range age | Overall, by camp & age group |
| 3 | Relationship to child | n/% by categories | Overall, by camp & age group |
| 4 | Date of arrival (if available) | Summarize/bar chart | Compare to dates of campaigns |
| 5 | Vaccine doses | n/% for each antigen, divide by # of children | Overall, by camp & age group |
| 6 | Campaign participation | n/% for each antigen | Overall, by camp & age group |
| 7 | Reasons for no vaccine, decision maker, who took child | n/% for each category, bar chart | Overall, by camp & age group |
| 9 | Vaccines at border entry | n/% yes/no | Overall, by camp & age group |
| 10 | Vaccines prior to entry | n/% by oral, injection, scar | Overall, by camp & age group |
| 11 | Vaccination card | n/% for each antigen | Overall, by camp & age group |
| 12 | Disease (malaria symptoms, measles, diphtheria) | n/% by disease/symptoms | Overall, by camp & age group |
| 13 | Map of vaccine coverage | GPS points for mapping each coverage of antigens | By camp & age group |
| **Dried blood spot collection** | | | |
| **#** | **Result** | **Analysis** | **Details** |
| 1 | Denominators | n/% pricked, refused, not present, failed: flowchart | Overall, by camp, day, team, age group |
| 2 | DBS vaccine immunity | n/% coverage for each antigen, table/graph | Overall, by camp & age group |

# **10. References**

1. Situation Report: Rohingya Refugee Crisis, Inter Sector Coordination Group. Cox’s Bazar, January 14 2018. <https://reliefweb.int/sites/reliefweb.int/files/resources/180115_weekly_iscg_sitrep_draft.pdf>
2. Bangladesh Rohingya Emergency Response, Early Warning Alert and Response System (EWARS), Annex W7. Ministry of Health and Family Welfare and World Health Organization. 20 February, 2018.
3. Bangladesh Rohingya Emergency Response, Diphtheria Outbreak, Update 09-02-2018. Ministry of Health and Family Welfare and World Health organization. 09 February, 2018.
4. http://www.who.int/neglected_diseases/diseases/yaws/en/
5. Weldon, W.C., M.S. Oberste, and M.A. Pallansch, Standardized Methods for Detection of Poliovirus Antibodies. Methods Mol Biol, 2016. **1387**: p. 145-76.
6. Scobie, H.M., et al., Tetanus Immunity among Women Aged 15 to 39 Years in Cambodia: a National Population-Based Serosurvey. Clin Vaccine Immunol, 2012. **23**: p. 546 –554
7. Hummel, Erdman, Heath, Bellini Baculovirus Expression of the Nucleoprotein Gene of Measles Virus and Utility of the Recombinant Protein in Diagnostic Enzyme Immunoassays. J. Clin Micro 1992; 30 (11): 2874-2880
8. Rogier, E., et al., Evaluation of Immunoglobulin G Responses to Plasmodium falciparum and Plasmodium vivax in Malian School Children Using Multiplex Bead Assay. Am J Trop Med Hyg, 2017. **96**(2): p. 312-318.
9. Hamlin, K.L., et al., Longitudinal Monitoring of the Development of Antifilarial Antibodies and Acquisition of Wuchereria bancrofti in a Highly Endemic Area of Haiti. PLoS Negl Trop Dis, 2012. **6**(12): p. e194.
10. Goodhew, B.E., et al., CT694 and pgp3 as Serological Tools for Monitoring Trachoma Programs. PLoS Negl Trop Dis. **6**(11): p. e1873 3
11. Cooley GM, Mitja O, Goodhew B, et al. Evaluation of Multiplex-Based Antibody Testing for Use in Large-Scale Surveillance for Yaws: a Comparative Study. Patel R, ed. Journal of Clinical Microbiology. 2016;54(5):1321-1325. doi:10.1128/JCM.02572-15.
12. Borrow R, Balmer P, Roper MH. The Immunological Basis for Immunization Series, Module 3: Tetanus. Update 2006. Geneva: WHO; 2006.
13. D.W. Scheifele, J.J. Ochnio. The Immunological Basis For Immunization Series, Module 2: Diphtheria. Update 2009 WHO, Geneva (2009)
14. Skendzel LP (1996) Rubella immunity. Defining the level of protective antibody. Am J Clin Pathol 106: 170–174. pmid:8712168
15. Lucchi, N.W., et al., PET-PCR method for the molecular detection of malaria parasites in a national malaria surveillance study in Haiti, 2011. Malar J, 2014. **13**: p. 462.

# **Annex D**

**Interviewer Script for Administration of**

**Caregiver Verbal Consent for Questionnaire and Fingerprick**

**(English)**


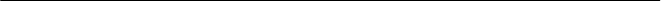


Hello. My name is________, and I am working with the Ministry of Health and Family Welfare and partners to learn more about diseases that are affecting the Rohingya community and how they can be prevented by vaccination. What we find during this assessment can help us with future prevention activities, like vaccination campaigns.

We have selected your house randomly and would like to ask you and 1–2 of your children to participate in the assessment.  For the selected children in the household (aged 6 months to less than 15 years), we will ask some questions about vaccination, and we may take a few small drops of blood (between 3 and 5 drops). This information will help the Ministry of Health and Family Welfare better understand if you and your community are protected against diseases such as measles, diphtheria, and malaria, and it will help to better plan for health, vaccination, and other services for your community in the future.

Whether you choose to participate in this study is entirely up to you. Participation is completely voluntary. You can also choose to stop your/your child’s participation at any time or withdraw from the study.

The information you share with us will be kept confidential.

The questions and fingerprick take about 10–15 minutes per child.

Do you have any questions? May I begin?
