# Supplementary material for: Vaccination coverage survey and seroprevalence among forcibly displaced Rohingya children, Cox's Bazar, Bangladesh, 2018: A cross-sectional study
Source: PLoS Med. 2020 Mar 31;17(3):e1003071. doi: 10.1371/journal.pmed.1003071 (PMC7108726; doi:10.1371/journal.pmed.1003071)
Supplement: S1 Questionnaire — (DOCX) [file pmed.1003071.s006.docx]

**Parent / Infant Caregiver Vaccination Questions**

শিশুর পিতামাতা / পরিচর্যাকারির টিকাদান সংক্রান্ত প্রশ্নাবলি

**Rohinyga population, Cox’s Bazar, Bangladesh**

রোহিঙ্গা জনগোষ্ঠি, কক্সবাজার, বাংলাদেশ

**000. General Information** সাধারণ তথ্য

| **ID** | **QUESTION**  প্রশ্ন | **RESPONSE**  **উত্তর** | **GO TO** যান |
| --- | --- | --- | --- |
| 001 | Team number  টীম নম্বর | ­­__ __ |  |
| 002 | Name of enumerator/interviewer  সাক্ষাতকার গ্রহনকারীর/ তথ্য সংগ্রহকারীর নাম |  |  |
| 003 | Date of interview  ইন্টারভিউ/ সাক্ষাতের তারিখ | **____ ____** / **____ ____** / 2018  DD দিন MM মাস/২০১৮ |  |
| 004 | Survey area  জরীপ এলাকা | 1. Kutupalong Registered Camp কতুপালং নিবন্ধিত ক্যাম্প  2. Nayapara Registered Camp নয়াপাড়া নিবন্ধিত ক্যাম্প  3. Makeshift / Informal Camps অস্থায়ী/অনিবন্ধিত ক্যাম্প |  |
| 005 | Block  ব্লক |  |  |
| 006 | Cluster  ক্লাস্টার |  |  |
| 007 | Household number  খানা নম্বর |  |  |
| 008 | Household serial number  খানা সিরিয়াল নম্বর |  |  |
| 009 | GPS coordinates  জিপিএস কোওর্ডিনেট |  |  |

Hello. My name is________, and I am working with the Ministry of Health and Family Welfare and partners to learn more about diseases that are affecting the Rohingya community and how they can be prevented by vaccination. What we find during this assessment can help us with future prevention activities, like vaccination campaigns.

We have selected your house randomly and would like to ask you and 1–2 of your children to participate in the assessment.  For the selected children in the household (aged 6 months to less than 15 years), we will ask some questions about vaccination, and we may take a few small drops of blood (between 3 and 5 drops). This information will help the Ministry of Health and Family Welfare better understand if you and your community are protected against diseases such as measles, diphtheria, and malaria, and it will help to better plan for health, vaccination, and other services for your community in the future.

Whether you choose to participate in this study is entirely up to you. Participation is completely voluntary. You can also choose to stop your/your child’s participation at any time or withdraw from the study.

The information you share with us will be kept confidential. The questions and fingerprick take about 10–15 minutes per child. Do you have any questions? May I begin?

আস্সালামুআলাইকুম/ আদাব। আমি ----------------------- । আমি বর্তমানে কক্সবাজার জেলায় অবস্থানকারী বলপূর্বক বাস্তুচ্যুত মিয়ানমার এর নাগরিকদের মধ্যে টিকাদান ও টিকা প্রদানের মাধ্যমে প্রতিরোধযোগ্য রোগ সম্পর্কিত গবেষণা করছি।

গবেষণার সারসংক্ষেপ ঃ

টিকাদান কর্মসূচী বাংলাদেশের একটি সফলতম কার্যক্রম। কক্সবাজার জেলায় অবস্থানকারী বলপূর্বক বাস্তুচ্যুত মিয়ানমার এর নাগরিকদের ৬ মাস হতে ১৫ বছর এর কম বয়সী শিশুদের কয়েকটি টিকাদান অভিযানে সময় টিকা প্রদানের কর্মসূচির মাধ্যমে প্রতিরোধ যোগ্য রোগসমুহ প্রতিরোধে টিকা প্রদান করা হচ্ছে। আমরা এ গবেষণার মাধ্যমে টিকা প্রদান পরবর্তী শিশুদের অর্জিত রোগ প্রতিরোধ ক্ষমতা এবং টিকাদান অভিযানে অংশ না নেয়ার কারণ সম্পর্কে জানতে আগ্রহী।

এ গবেষণা কার্যক্রমে কেন আপনাকে নির্বাচিত করা হলো?

আমাদের গবেষণা কার্যক্রম সেই সকল শিশুদের উপর পরিচালিত হচ্ছে, যাদের বয়স ৬ মাস থেকে ১৫ বছরের মধ্যে, বলপূর্বক বাস্তুচ্যুত মিয়ানমার এর নাগরিক এবং কক্সবাজার জেলায় অবস্থানকারী । আপনি এ রকমের একজন শিশুর অভিভাবক হওয়া তে আপনাকে সাক্ষাৎকারের জন্য নির্বাচিত করা হয়েছে।

অংশ গ্রহনের ধরণঃ

আপনি যদি আমাদের গবেষণা কার্যক্রমে অংশগ্রহনের সম্মতি প্রদান করেন, তাহলে আমরা আপনার একটি সাক্ষাৎকার গ্রহণ করব। সাক্ষাৎকারটি দিতে আপনার ১৫ মিনিটের মতো সময় লাগবে। সাক্ষাৎকারে আমরা আপনার আর্থ-সামাজিক অবস্থা, শিশুর টিকা প্রদান সম্পর্কিত তথ্য এবং শিশুর টিকা প্রদানের সমস্যা সম্পর্কিত কিছু প্রশ্ন করব। এ ছাড়া শিশুর নিকট হকে ৩-৪ ফোঁটা রক্ত সংগ্রহকরব।

সাক্ষাৎকার চলাকালে যে কোনো সময়ে আপনি ইচ্ছা করলে সাক্ষাৎকার প্রদান থেকে নিজেকে বিরত রাখতে পারেন।

অংশ গ্রহনের ঝুঁকি এবং প্রাপ্ত সুবিধাসমুহঃ

আমাদের গবেষণা কার্যক্রমে অংশ গ্রহনে আপনার / আপনার শিশুর অথবা আপনার পরিবারের কোন ঝুঁকি নেই। আমাদের গবেষণা কার্যক্রমে অংশগ্রহন করে আপনি কোনো প্রকার ব্যক্তিগত অথবা আর্থিক সুবিধা পাবেন না। শিশুর রক্ত সংগ্রহের সময় সামান্য ব্যথা লাগতে পারে, যা সাময়িক, এ সংক্রান্ত অন্য জটিলতা হলে আমরা প্রয়োজনীয় ব্যবস্থা নিব।

ব্যক্তিগত পরিচিতি ও প্রদত্ত তথ্যের গোপনীয়তাঃ

আমরা আপনার / আপনার শিশুর ব্যক্তিগত পরিচিতি ও প্রদত্ত তথ্যের গোপনীয়তা রক্ষা করার প্রতিশ্রুতি দিচ্ছি। আপনি ইচ্ছা করলে তথ্য প্রদান থেকে নিজেকে বিরত রাখতে পারেন, সে ব্যাপারে আপনার সম্পূর্ণ স্বাধীনতা আছে।

আমরা নিশ্চয়তা দিচ্ছি যে, তথ্য-উপাত্ত বিশ্লেষণের সময় এবং রিপোর্ট প্রকাশের সময় আপনার / আপনার শিশুর অথবা আপনার পরিবারের নাম ও ঠিকানা সম্পূর্ণ রূপে গোপন রাখা হবে। সকল সাক্ষাৎকার ও তথ্য-উপাত্ত আমাদের অফিসে তালাবদ্ধ থাকবে।

তথ্য-উপাত্তের ভবিষ্যৎ ব্যবহার ঃ

আমরা নিশ্চয়তা দিচ্ছি যে, ভবিষ্যতে যে কোনো সময়ে আপনার প্রদত্ত তথ্য-উপাত্ত পুনঃব্যবহার করা হলে আপনার পরিচিতি অবশ্যই গোপন রাখা হবে।

তথ্য প্রদানে অসম্মতি জ্ঞাপনের স্বাধীনতা ঃ

এই গবেষণা কার্যক্রমে আপনার / আপনার শিশুর অংশগ্রহন নিজের ইচ্ছার উপর নির্ভরশীল। আপনি ইচ্ছা করলে তথ্য প্রদান থেকে নিজেকে বিরত রাখতে পারেন, সে ব্যাপারে আপনার সম্পূর্ণ স্বাধীনতা আছে। তথ্য প্রদান থেকে বিরত থাকলে, আপনি কোনো রকম আইনী জটিলতায় পড়বেন না।

ক্ষতিপূরণ প্রাপ্তির অধিকার ঃ

এই গবেষণা কার্যক্রমে আপনার অংশগ্রহন করে আপনি কোনোরকম ব্যক্তিগত অথবা আর্থিক ক্ষতির সম্মুখীন হবেন না বিধায়, এই গবেষণাকার্যক্রম থেকে, কোনো রকম ব্যক্তিগত অথবা আর্থিক ক্ষতিপূরণ দাবী করতে পারবেন না। তবে শিশুর রক্ত সংগ্রহের সংক্রান্ত কোনো জটিলতা হলে আমরা প্রয়োজনীয় ব্যবস্থা নিব।

এই গবেষণা কার্যক্রম সম্পর্কে আরও কিছ ুজিজ্ঞাসা থাকলে আইইডিসিআর বরাবরে যোগাযোগ করবেন।আপনি যদি উপরোক্ত তথ্যাবলী সম্পূর্ণরূপে বুঝে, আমাদের গবেষণা কার্যক্রমে অংশগ্রহনের সম্মত থাকেন তবে আপনাকে স্বাক্ষর/ টিপসহি প্রদান করতে হবে। আমরা এই সম্মতিপত্রের এক কপি আপনাকে দিয়ে যাবো।

গবেষণা কার্যক্রমে অংশগ্রহনের জন্য আপনাকে ধন্যবাদ।

আমি কি সাক্ষাতকারটি শুরু করতে পারি?

IF CONSENT REFUSED: Please ensure that Team Leader has explained clearly the objectives of the survey. If the head of household/respondent still refuses, **go to end of questionnaire**.

সম্মতি না প্রদান করলে- গবেষণার উদ্দেশ্য সম্পর্কে পরিস্কারভাবে জানাতে হবে। এর পরও খানা প্রধান / উত্তরদাতা সম্মতি প্রদান না করলে- প্রশ্ন পত্রের শেষে যান।

First, Can you share with me any documents and cards you have received related to the vaccinations and health facility or hospital visits for your children? *Interviewer should show what cards he/she is interested in seeing.*

প্রথমত, আপনার সন্তানদের টিকাদান, স্বাস্থ্য ব্যাবস্থা বা হাসপাতালে ভর্তি সম্পর্কিত কোনও দলিল এবং কার্ড থাকলে আমাকে দেখতে দিন। সাক্ষাতকারগ্রহনকারির দেখানো উচিত কোন কার্ডগুলি তিনি দেখতে আগ্রহী।.

**100. Vaccination of Child Aged 6 months to 6 years**

**শিশুকে টিকাদান- ৬ মাস থেকে ৭ বছর বয়স পর্যন্ত**

I would now like to ask you about your child’s vaccination history.

আমি এখন আপনাকে আপনার সন্তানের টিকা সম্পর্কে কিছু জিজ্ঞাসা করতে চাই।

*Ask child’s name and substitute “this child” with the name of the child in this section.*

সন্তানের নাম জিজ্ঞাসা করুন এবং এই বিভাগে "এই শিশুর" স্থলে সন্তানের নাম ব্যবহার করুন।

| **ID** | **QUESTION**  প্রশ্ন | **RESPONSE**  **উত্তর** | **GO TO**  যান |
| --- | --- | --- | --- |
| 101 | Child #  শিশুর নম্বর | ___ ___ ___ ___ ___ ___ |  |
| 102 | Child age  শিশুটির বয়স | ______ months / years *(circle)*  *মাস / বছর (বৃত্ত আঁকুন)*  88. Refused to answer উত্তর দিতে রাজি না |  |
| 103 | Is the child present?  *আপনার শিশুটি এখানে উপস্থিত ?* | 1. Yes হ্যাঁ  2. No না |  |
| 104 | What is your relationship with the selected child?  নির্বাচিত শিশুর সঙ্গে আপনার সম্পর্ক কি?  *The respondent may consult with his/her spouse, children, neighbors, etc.*  উত্তরদাতআ তার স্বামী/ স্ত্রী, শিশু, প্রতিবেশী, ইত্যাদির সঙ্গে পরামর্শ করতে পারবেন | 1. Mother মা  2. Father বাবা  3. Grandmother দাদী/ নানী  4. Grandfather দাদা/নানা  5. Aunt খালা/ ফুপু/ চাচী/কাকী/মামী  6. Uncle খালু/ফুপা/ চাচা/কাকা/মামা  7. Sister বোন  8. Brother ভাই  9. Other relative অন্যান্য অন আত্মীয়  10. Other অন্যান্য  88. Refused to answer  উত্তর দিতে অস¦ীকৃতি জানানো |  |
| 105 | Did this child arrive in Bangladesh together with the family?  শিশুটি কি পরিবার সহ বাংলাদেশে এসেছিল ? | 1. Yes হ্যাঁ ------------------------------------------->  2. No না  88. Refused to answer --------------------------->  উত্তর দিতে অস¦ীকৃতি জানানো  99. Don’t know জানি না ---------------------------> | Q107  Q107  Q107 |
| 106 | If no, (the child arrived earlier or later), when did the child arrive in Bangladesh?  উত্তর না হলে (শিশুটি আগে/পরে আসলে) শিশুটি কবে বাংলাদেশে এসেছিল ?  *Dates can be calculated based on Kurbani Eid (01 Sep 2017). Estimate the number of lunar months after Eid the child arrived.*  *কুরবানির ঈদ (১ সেপ্টেম্বর, ২০১৭) থেকে তারিখ গণনা করা যেতে পারে। ঈদের পর থেকে চন্দ্রমাস গণনা করুন।* | ___ ___ lunar cycles after EID  ঈদের **____** চন্দ্রমাস পর  88. Refused to answer উত্তর দিতে অস্বীকার  99. Don’t know জানি না |  |
| 107 | Who takes the decision to vaccinate or not vaccinate this child?  আপনার শিশুর টিকা দেয়া / না দেয়া সিদ্ধান্ত কে নেয় ?  *Do not reach choices. Mark all that are mentioned.*  পছন্দগুলো বলবনে না। যাদের নাম বলা হয় সবগুলো লিপিবদ্ধ করুন ন। | 1. Mother মা  2. Father বাবা  3. Sister বোন  4. Brother ভাই  5. Other female relative  অন্য কোন মহিলা আত্মীয়  6. Other male relative  অন্য কোন পুরুষ আত্মীয়  7. Other অন্যান্য  8. The child by himself or herself শিশু নিজেই  9. No one কেউ না  88. Refused to answer উত্তর দিতে অস্বীকার  99. Don’t know জানি না |  |
| *If card is available, use date on vaccination card to answer questions. If no date is written on the card(s) or if card is missing, ask corresponding questions. For these questions, it may be helpful to use the event of Kurbani Eid, 01 Sep 2017, (or their arrival date if close to Kurbani Eid) to associate the first 2 campaigns (MR 1^st^ round and OCV 1^st^ round). As a reference, children under 2 years of age should have received injections in the thigh. Children 2 years and older should have received injections in the arm.*  *টিকাদান কার্ডটি থাকলে, টিকার তারিথ কার্ডে উল্লিখিত তারিখ লিখুন। টিকাদান কার্ডে তারিখ না থাকলে অথবা কার্ডটি হারিয়ে গেলে , নিচের টিকার তারিখ সম্পর্কিত প্রশ্নগুলো করুন।*  এই প্রশ্নগুলি প্রথম দুটো টিকাদান ক্যাম্পেইন (এমআর ১ম রাউন্ড এবং ওসিভি ১ম রাউন্ড) এর সাথে সংযুক্ত করার জন্য কোরবানি ঈদ ০১ সেপ্টেম্বর ২০১৭, (অথবা তাদের আগমনের তারিখ যদি কোরবানি ঈদের কাছাকাছি হয়) ব্যবহার করা যেতে পারে।  একটি রেফারেন্স হিসাবে বলা যায়, ২ বছরের কম বয়সী শিশুদের ঊরুতে এবং ২ বছর এবং তার উপরের বয়সের শিশুদের বাহুতে ইনজেকশন পাওয়া উচিত। | | | |
|  | How many times did this child take diphtheria vaccine?  এই শিশুটি কতবার ডিপথেরিয়ার টিকা নিয়েছিল?  *This question is asked to guide the next few questions and will not be recorded.*  পরবর্তী কয়েকটি প্রশ্নের দিক নির্দেশনার এই প্রশ্নটি করা হবে কিন্তু রেকর্ড করা হবে না। |  |  |
| 108 | About a month ago, there was a diphtheria vaccination campaign that included **one diphtheria injection** and **oral polio drops**. Did this child receive an injection and oral drops? This child might have had **pain on touch** and maybe slight fever, which is normal. You may have received a **vaccination card** as well.  *(10–29 March 2018:*  *Penta+OPV, 3^rd^ round for 6weeks–<7years)*  প্রায় এক মাস আগে একটি ডিপথেরিয়া টিকাদান অভিযান হয়েছিল, যেখানে একটি ডিপথেরিয়া ইনজেকশন ও পোলিও ড্রপ মুখে খাওয়ানো হয়েছিল।  আপনার শিশুটি কি সেখানে একটি ডিপথেরিয়া ইনজেকশন ও একটি পোলিও ড্রপ মুখে খেয়েছিল্ ?  টিকাদান পরবর্তী সময়ে, সামান্য ব্যাথা অথবা সামান্য জ্বর হতে পারে, যা স্বাভাবিক। আপনি সেখানে একটি টিকাদান কার্ডও পেতে পারেন  (মার্চ, ২০১৮: পেন্টা + ওপিভি ৩য় রাউন্ড- ৬ সপ্তাহ- অনুর্ধ্ব ৭ বছর বয়সী শিশুর জন্য) | 1. Yes, confirmed by date in vaccination card --->  হ্যাঁ, টিকা কার্ডের তারিখ অনুযায়ী নিশ্চিত  2. Yes, by recall ----------------------------------->  হ্যাঁ, সাক্ষাতকারী প্রদানকারী মনে করে বললেন  3. No না  88. Refused to answer উত্তর দিতে রাজি না ------>  99. Don’t know জানি না ---------------------------> | Q110  Q110  Q111  Q111 |
| 109 | What are the reasons why the child did not get vaccinated?  শিশুটির টিকা না পাওয়ার কারণগুলো কি ?  *Instructions:*  *The respondent should be prompted: ‘Are there any other reasons?’ until they have mentioned all reasons.*  নির্দেশাবলী:  উত্তরদাতাকে অনুরোধ করা উচিত: *’আরও কোন কারণ আছে কিনা’?*  *Do not read choices. Mark all that are mentioned.*  পছন্দগুলি পড়বেন না । সেগুলো উল্লেখ করবে সেসব চিহ্নিত করুন। | 1. Caregiver was unaware of the vaccination campaign  পরিচর্যাকারি টিকাদান ক্যাম্পেইন সম্পর্কে সচেতন ছিলনা  2. Caregiver thinks that child received all required vaccines  পরিচর্যাকারি মনে করে যে শিশুটি সব প্রয়োজনীয় টিকা গ্রহন করেছে  3. Caregiver does not think vaccines are necessary  পরিচর্যাকারি চান না যে শিশুটি টিকা গ্রহন করুক  4. Caregiver is fearful of vaccines or fearful of multiple injections  পরিচর্যাকারী অনেকগুলো ইনজেকশন এক সঙ্গে দিতে হবে, তাই ভয় পেয়েছিলেন  5. Caregiver or child is fearful about not going to heaven or being converted to Christianity  পরিচর্যাকারী জান্নাতে না যেতে পারা অথবা খ্রীস্ট ধর্মে রূপান্তরিত হওয়ার ভয় পেয়েছেন  6. Female caregiver or child not comfortable leaving the house  মহিলা পরিচর্যাকারী অথবা শিশুটির পক্ষে ঘরটি ছেড়ে যাওয়া সম্ভবপর ছিল না    7. Female caregiver or child not comfortable taking vaccine from a male vaccinator  মহিলা পরিচর্যাকারী অথবা শিশুটির নিকট পুরুষ টিকাদানকারী গ্রহনযোগ্য ছিল না  8. The child got fever/swelling after last vaccination  শিশুটির শেষ টিকা গ্রহনের পর জ্বর / টিকার স্থানের ফুলে গিয়েছিল  9. No one was available to take the child to vaccination.  শিশুকে টিকা দিতে নিয়ে যাওয়ার জন্য কেউ ছিলনা  10. Child was not available (e.g., not at home, sick) at time of vaccination  শিশুটি বাসায় ছিল না / অসুস্থ ছিল  11. Child was afraid of needles/pain  শিশুটি ইনজেকশন/ ব্যাথার ভয় পেয়েছিল  12. Father or head of household does not allow  শিশুর পিতা / পরিবার প্রধান অনুমতি দেয়নি  13. Vaccinator treated caregiver or child poorly  টিকাদান কারী শিশু/ পরিচর্যাকারীর সাথে ভালো ব্যবহার করেননি  14. Other অন্যান্য  88. Refused to answer উত্তর দিতে অস্বীকার  99. Don’t know জানি না | Q111 |
| 110 | Who took the child to get vaccinated?  টিকা দিতে কে শিশুকে নিয়ে গিয়েছিল?  *Do not reach choices. Mark only one.*  পছন্দগুলো পড়িবেন না, শুধুমাত্র একটিতে টিক চিহ্ন দিন। | 1. Mother মা  2. Father বাবা  3. Sister বোন  4. Brother ভাই  5. Other female relative  অন্য কোন মহিলা আত্মীয়  6. Other male relative অন্য কোন পুরুষ আত্মীয়  7. Neighbor/friend প্রতিবেশী / বন্ধু  8. Social mobilizer সমাজসেবী  9. Vaccinated at school  স্কুলে টিকা দেয়া হয়েছিল  10. Vaccinated at madrassa  মাদ্রাসায় টিকা দেয়া হয়েছিল  11. Child went by him/her self শিশু নিজে গিয়েছে  12. Other অন্যান্য ---------------------  88. Refused to answer উত্তর দিতে অস্বীকার  99. Don’t know জানি না |  |
| 111 | Before the last campaign, there was another diphtheria vaccination campaign that included **one diphtheria injection** and **oral polio drops**. Did this child receive an injection and oral drops? This child might have had **pain on touch** and maybe slight fever, which is normal. You may have received a **vaccination card** as well.  গত টিকাদান অভিযান এর আগে, আরও একটি ডিপথেরিয়া টিকাদান অভিযান হয়েছিল, যেখানে একটি ডিপথেরিয়া ইনজেকশন ও পোলিও ড্রপ মুখে খাওয়ানো হয়েছিল।  আপনার শিশুটি কি সেখানে একটি ডিপথেরিয়া ইনজেকশন ও একটি পোলিও ড্রপ মুখে খেয়েছিল্ ?  টিকাদান পরবর্তী সময়ে, সামান্য ব্যাথা অথবা সামান্য জ্বর হতে পারে, যা স্বাভাবিক। আপনি সেখানে একটি টিকাদান কার্ডও পেতে পারেন  *(27 January–10 February 2018:*  *Penta+OPV, 2^nd^ round for 6weeks–<7years)*  (২৭ জানুয়ারী থেকে ১০ ফেব্রুয়ারী, ২০১৮: পেন্টা + ওপিভি ২য় রাউন্ড- ৬ সপ্তাহ- অনুর্ধ্ব ৭ বছর বয়সী শিশুর জন্য) | 1. Yes, confirmed by date in vaccination card  হ্যাঁ, টিকা কার্ডের তারিখ অনুযায়ী নিশ্চিত  2. Yes, by recall হ্যাঁ,স্মরন দ্বারা  3. No না  88. Refused to answer উত্তর দিতে রাজি না  99. Don’t know জানি না |  |
| 112 | And before that campaign, there was another **diphtheria** vaccination campaign that included **two injections, one in each thigh or arm,** and **oral polio drops**. Did this child receive the two injections and oral drops?  This child might have had **pain on touch** and maybe slight fever, which is normal. You may have received a **vaccination card** as well.  *(12–31 December 2017:*  *Penta+PCV+OPV, 1^st^ round for 6weeks–<7years)*  এবং সেই ক্যাম্পেইনের আগে, আরও একটি ডিপথেরিয়া টিকাদান অভিযান হয়েছিল, যেখানে প্রত্যেক উরু অথবা বাহুতে দুইটি ইনজেকশন ও পোলিও ড্রপ মুখে খাওয়ানো হয়েছিল।  আপনার শিশুটি কি সেখানে দুইটি ইনজেকশন ও একটি পোলিও ড্রপ মুখে খেয়েছিল্ ?  টিকাদান পরবর্তী সময়ে, সামান্য ব্যাথা অথবা সামান্য জ্বর হতে পারে, যা স্বাভাবিক। আপনি সেখানে একটি টিকাদান কার্ডও পেতে পারেন  (১২-৩১ ডিসেম্বর, ২০১৭: পেন্টা +পিসিভি+ ওপিভি ১ম রাউন্ড- ৬ সপ্তাহ - অনুর্ধ্ব ৭ বছর বয়সী শিশুর জন্য) | 1. Yes, confirmed by date in vaccination card  হ্যাঁ, টিকা কার্ডের তারিখ অনুযায়ী নিশ্চিত  2. Yes, by recall হ্যাঁ,স্মরন দ্বারা  3. No না  88. Refused to answer উত্তর দিতে রাজি না  99. Don’t know জানি না |  |
|  | How many times did this child take measles vaccine?  এই শিশুটি কতবার হামের টিকা নিয়েছে?  *This question is asked to guide the next few questions and will not be recorded.*  এই প্রশ্নটি পরবর্তী কয়েকটি প্রশ্নের দিক নির্দেশনার জন্য বলা হয়েছে এবং এটি রেকর্ড করা হবে না। |  |  |
| 113 | Before the diphtheria vaccination campaign, there was a vaccination campaign that included **one** **injection for measles and NO oral drops**. Did this child receive the injection? You may have received a **vaccination card** as well.  ডিপথেরিয়া টিকাদান অভিযান এর আগে আরও একটি অভিযান হয়েছিল, যেখানে একটি হামের টিকার ইনজেকশন দেয়া হয়েছিল, কোনো পোলিও ড্রপ মুখে খাওয়ানো হযনি।  আপনার শিশুটি কি টিকা পেয়েছিল ?  আপনি সেখানে একটি টিকাদান কার্ডও পেতে পারেন ।  *(18 November–05 December 2017:*  *MR, 2^nd^ round for 6months–<15years)*  (১৮ নভেম্বর থেকে ৫ ডিসেম্বর, ২০১৭ এম আর ২য় রাউন্ড- ৬ মাস- অনুর্ধ্ব ১৫ বছর বয়সী শিশুর জন্য) | 1. Yes, confirmed by date in vaccination card  হ্যাঁ, টিকা কার্ডের তারিখ অনুযায়ী নিশ্চিত  2. Yes, by recall হ্যাঁ,স্মরন দ্বারা  3. No না  88. Refused to answer উত্তর দিতে রাজি না  99. Don’t know জানি না |  |
| 114 | There was another measles vaccination campaign a few weeks after Kurbani Eid that included cutting of the **Vitamin A red pill** for young children, **measles injection** and **oral polio drops**. Did this child receive the injection and oral drops? You may have received a **vaccination card** as well.  কুরবানির ঈদেব কয়েক সপ্তাহ পরে আরও একটি হামের টিকাদান অভিযান হয়েছিল, যেখানে একটি ভিটামিন এ এর লাল পিল, একটি হামের টিকার ইনজেকশন দেয়া হয়েছিল ও পোলিও ড্রপ মুখে খাওয়ানো হয়েছিল ।  আপনার শিশুটি কি টিকাগুলো পেয়েছিল ?  আপনি সেখানে একটি টিকাদান কার্ডও পেতে পারেন ।  *(16 September–03 October 2017:*  *OPV only for 0–<6months*  *OR MR+OPV+Vitamin A, 1^st^ round for 6months–<5years OR MR only for 5years–<15years)*  (১৬ সেপ্টেম্বর থেকে ০৩ অক্টোবর, ২০১৭:  ওপিভি-০ থেকে অনুর্ধ্ব ৬ মাস  ভিটামির এ+ এম আর + ওপিভি- ৬ মাস- অনুর্ধ্ব ৫ বছর বয়সী শিশুর জন্য  এম আর ১ম রাউন্ড ৫ বছর -অনুর্ধ্ব ১৫ বছর বয়সী শিশুর জন্য) | 1. Yes, confirmed by date in vaccination card  হ্যাঁ, টিকা কার্ডের তারিখ অনুযায়ী নিশ্চিত  2. Yes, by recall হ্যাঁ,স্মরন দ্বারা  3. No না  88. Refused to answer উত্তর দিতে রাজি না  99. Don’t know জানি না |  |
|  | How many times did this child take oral cholera vaccine from a small bottle?  আপনার শিশুকে কত বার ছোট একটি বোতল থেকে কলেরা টিকা মুখে খাওয়ানো হয়েছিল ?  *This question is asked to guide the next few questions and will not be recorded.*  এই প্রশ্নটি পরবর্তী কয়েকটি প্রশ্নের দিক নির্দেশনার জন্য বলা হয়েছে এবং এটি রেকর্ড করা হবে না। |  |  |
| 115 | After the Vitamin A / measles campaign, there was a vaccination campaign that included **oral cholera vaccine in a small bottle for both adults and children** (from 1 year and above). Did this child receive the oral vaccine? You may have received **soap** as well.  ভিটামিন এ ও হামের টিকাদান অভিযান এর পরে একটি অভিযান হয়েছিল, যেখানে একটি ছোট একটি বোতল থেকে কলেরা টিকা বড় ও শিশুদের ( ১বছর ও তদুর্ধ্ব) খাওয়ানো হয়েছিল।  আপনার শিশুটি কি কলেরা টিকা পেয়েছিল ?  আপনি সেখানে একটি সাবানও পেতে পারেন।  *(10–18 October 2017:*  *OCV, 1^st^ round for ≥1year)*  (১০-১৮ অক্টোবর, ২০১৭: ওসিভি ১ম রাউন্ড- ১বছর ও তদুর্ধ্ব) | 1. Yes হ্যাঁ  2. No না  88. Refused to answer উত্তর দিতে রাজি না  99. Don’t know জানি না |  |
| 116 | After the oral cholera vaccine campaign that included soap, there was another vaccination campaign that included **oral cholera vaccine in a small bottle** but only for children older than 1 but less than 5 years old. Did this child receive the oral vaccine?  কলেরা টিকাদান অভিযান, যেখানে একটি সাবান দেয়া হয়েছিল, তার পরে একটি অভিযান হয়েছিল, যেখানে একটি ছোট একটি বোতল থেকে কলেরা টিকা ১-৫বছর বয়সী শিশুদের খাওয়ানো হয়েছিল।  আপনার শিশুটিকে কি কলেরা টিকা খাওয়ানো হয়েছিল।  *(04–09 November 2017:*  *OCV+OPV, 2^nd^ round for 1year–<5years*  *OR OPV only for <1year)*  (০৪-০৯ নভেম্বর, ২০১৭: ওসিভি + ওপিভি ২য় রাউন্ড- ১ বছর -৫বছর  এবং শুধুমাত্র ওপিভি অনুর্ধ্ব ১ বছর বয়সী শিশু) | 1. Yes হ্যাঁ  2. No না  88. Refused to answer উত্তর দিতে রাজি না  99. Don’t know জানি না |  |
| 117 | When you and your family entered Bangladesh, did this child take any vaccination at the border, either injections or oral polio drops?  আপনি এবং আপনার পরিবার যখন বাংলাদেশে এসেছিলেন, তখন সীমান্তে আপনার শিশু কি কোনো ইনজেকশন অথবা মুখে পোলিও টিকা খাওয়ানো হয়েছিলো? | 1. Yes, confirmed by date in vaccination card  হ্যাঁ, টিকা কার্ডের তারিখ অনুযায়ী নিশ্চিত  2. Yes, by recall হ্যাঁ,স্মরন দ্বারা  3. No না  88. Refused to answer উত্তর দিতে রাজি না  99. Don’t know জানি না |  |
| 118 | Can you share any additional vaccination cards or medical records related to this child?  আপনি কি এই শিশুর সাথে সম্পর্কিত কোনও অতিরিক্ত টিকাদান কার্ড বা মেডিকেল রেকর্ড দেখাতে পারেন?  *If there are any additional vaccination cards that do not correspond to campaign doses, vaccination at the border or vaccination for being a diphtheria contact, indicate which vaccines were received.*  যদি কোনো অতিরিক্ত টিকাদান কার্ড থাকে যা ক্যাম্পেইন ডোজ, সীমান্তে টিকা বা ডিপথেরিয়ার কারনে টিকার সাথে সম্পর্কিত নয় তাহলে, কোন টিকা তা নির্দেশ করুন। | 1. OPV, confirmed by date in vaccination card  ওপিভি, টিকা কার্ডের তারিখ অনুযায়ী নিশ্চিত  2. PCV, confirmed by date in vaccination card  পিসিভি টিকা কার্ডের তারিখ দ্বারা নিশ্চিত  3. Penta, confirmed by date in vaccination card  পেন্টা টিকা কার্ডের তারিখ অনুযায়ী নিশ্চিত  4. Td, confirmed by date in vaccination card  টিদি, টিকা কার্ডের তারিখ দ্বারা নিশ্চিত  5. MR, confirmed by date in vaccination card  এম.আর.টিকা কার্ডের তারিখ অনুযায়ী নিশ্চিত  6. BCG, confirmed by date in vaccination card  বিসিজি, টিকাদান কার্ড থেকে নিশ্চিত  7. IPV, confirmed by date in vaccination card  আইপিভি, টিকাদান কার্ড থেকে নিশ্চিত  8. Other অন্যান্য  9. No other vaccination card available  অন্য কোন টিকা কার্ড পাওয়া যায় না |  |
| 119 | When this child lived in Myanmar, did this child ever take oral polio drops. Polio is a vaccine given by dropping liquid into a child’s mouth. Oral polio drops are different from Vitamin A drops, which usually are red or blue pills.  যখন এই শিশুটি মায়ানমারে বসবাস করত, তখন এই শিশুটি কি কখনো পোলিও টিকা বা মৌখিক পোলিও ড্রপ গ্রহন করেছে? পোলিও একটি তরল টিকা যা শিশুর মুখের মধ্যে ড্রপ দ্বারা দেওয়া হয়। | 1. Yes, confirmed by date in vaccination card  হ্যাঁ, টিকা কার্ডের তারিখ অনুযায়ী নিশ্চিত  2. Yes, by recall হ্যাঁ,স্মরন দ্বারা  3. No না  88. Refused to answer উত্তর দিতে রাজি না  99. Don’t know জানি না |  |
| 120 | When this child lived in Myanmar, did this child ever take any vaccination through injection?  যখন এই শিশু মায়ানমারে বসবাস করত, তখন কি এই শিশুটি ইনজেকশন এর মাধ্যমে কোনো টিকা নিয়েছে ? | 1. Yes, confirmed by date in vaccination card  হ্যাঁ, টিকা কার্ডের তারিখ অনুযায়ী নিশ্চিত  2. Yes, by recall হ্যাঁ,স্মরন দ্বারা  3. No না  88. Refused to answer উত্তর দিতে রাজি না  99. Don’t know জানি না |  |
| 121 | *Does the child have a BCG scar? Check the child’s upper right and left arms. (Do not ask the caregiver.)*  এই শিশুটির কি *বিসিজি টিকার চিহ্ন* আছে? শিশুর ডান এবং বাম বাহুর উপরে পরীক্ষা করুন। (পরিচর্যাকারিকে জিজ্ঞাসা করবেন না।) | 1. Yes হ্যাঁ  2. No না  3. Not sure if scar is from BCG  নিশ্চিত না স্কারটি বিসিজির কিনা  99. Child not available শিশুটি উপস্থিত নাই |  |
| 122 | In the past two weeks, has this child had a fever without rash?  বিগত ২ সপ্তাহে কি শিশুর র‌্যাশ ছাড়া জ্বর হয়েছিলো ? | 1. Yes হ্যাঁ  2. No না  88. Refused to answer উত্তর দিতে রাজি না  99. Don’t know জানি না |  |
| 123 | After arrival in Bangladesh, has this child had measles?  বাংলাদেশে আগমনের পর, শিশুর কি কখনো হাম হয়েছিলো ? | 1. Yes, confirmed by health facility document  হ্যাঁ, স্বাস্থ্য কেন্দ্রের দলিল অনুযায়ী নিশ্চিত  2. Yes, verbal report that the child was diagnosed at a clinic  হ্যাঁ, কোনো ক্লিনিকে মৌখিকভাবে নিশ্চিত  3. Yes, verbal report that the child was diagnosed by a local healer  হ্যাঁ, কোনো স্থানীয় পল্লী চিকিসক কর্তৃক মৌখিকভাবে নিশ্চিত  4. Yes, verbal report but did not seek diagnosis  হ্যাঁ, মৌখিক রিপোর্ট কিন্তু ওই রোগের জন্য যায়নি  5. No না  88. Refused to answer উত্তর দিতে রাজি না  99. Don’t know জানি না |  |
| 124 | After arrival in Bangladesh, has this child had diphtheria?  বাংলাদেশে আগমনের পর, শিশুর কি কখনো ডিপথেরিয়া হয়েছিলো ? | 1. Yes, confirmed by health facility document  হ্যাঁ, স্বাস্থ্য কেন্দ্রের দলিল অনুযায়ী নিশ্চিত  2. Yes, verbal report that the child was diagnosed at a clinic  হ্যাঁ, কোনো ক্লিনিকে মৌখিকভাবে নিশ্চিত  3. Yes, verbal report that the child was diagnosed by a local healer  হ্যাঁ, কোনো স্থানীয় পল্লী চিকিসক কর্তৃক মৌখিকভাবে নিশ্চিত  4. Yes, verbal report but did not seek diagnosis  হ্যাঁ, মৌখিক রিপোর্ট কিন্তু ওই রোগের ক্লিনিকে জন্য যায়নি  5. No না  88. Refused to answer উত্তর দিতে রাজি না  99. Don’t know জানি না |  |

**200. Vaccination of Child Aged 7years to 14years শিশুকে টিকাদান ৭-১৫ বছর বয়স পর্যন্ত**

I would now like to ask you about your child’s vaccination history.

আমি এখন আপনার সন্তানের টিকা সম্পর্কে আপনাকে জিজ্ঞাসা কিছু করতে চাই।

*Ask child’s name and substitute “this child” with the name of the child in this section.*

সন্তানের নাম জিজ্ঞাসা করুন এবং এই বিভাগে "এই শিশুর" স্থলে সন্তানের নাম ব্যবহার করুন।

| **ID** | **QUESTION**  প্রশ্ন | **RESPONSE**  **উত্তর** | **GO TO**  যান |
| --- | --- | --- | --- |
| 201 | Child #  **শিশুর নম্বর** | ___ ___ ___ ___ ___ ___ |  |
| 202 | Child age  শিশুটির বয়স | ______ months / years *(circle)*  *মাস/বছর (বৃত্ত আঁকুন)*  88. Refused to answer উত্তর দিতে রাজি না |  |
| 203 | Is the child present?  *আপনার শিশুটি এখানে উপস্থিত ?* | 1. Yes হ্যাঁ  2. No না |  |
| 204 | What is your relationship with the selected child?  নির্বাচিত শিশুর সঙ্গে আপনার সম্পর্ক কি?  *The respondent may consult with his/her spouse, children, neighbors, etc.*  উত্তরদাত তার স্বামী/ স্ত্রী, শিশু, প্রতিবেশী, ইত্যাদির সঙ্গে পরামর্শ করতে পারবেন | 1. Mother মা  2. Father বাবা  3. Grandmother দাদী/ নানী  4. Grandfather দাদা/নানা  5. Aunt খালা/ ফুপু/ চাচী/কাকী/মামী  6. Uncle খালু/ফুপা/ চাচা/কাকা/মামা  7. Sister বোন  8. Brother ভাই  9. Other relative অন্যান্য অন আত্মীয়  10. Other অন্যান্য  88. Refused to answer উত্তর দিতে অস্বীকার |  |
| 205 | Did this child arrive in Bangladesh together with the family?  শিশুটি কি পরিবার সহ বাংলাদেশে এসেছিল ? | 1. Yes হ্যাঁ ---------------------------------------->  2. No না  88. Refused to answer  উত্তর দিতে অস¦ীকৃতি জানানো ----------->  99. Don’t know জানি না -------------------------> | Q207  Q207  Q207 |
| 206 | If no, (the child arrived earlier or later), when did the child arrive in Bangladesh?  উত্তর না হলে (শিশুটি আগে/পরে আসলে) শিশুটি কবে বাংলাদেশে এসেছিল ?  *Dates can be calculated based on Kurbani Eid (01 Sep 2017). Estimate the number of lunar months after Eid the child arrived.*  *কুরবানির ঈদ (১ সেপ্টেম্বর, ২০১৭) থেকে তারিখ গণনা করা যেতে পারে। ঈদের পর থেকে চন্দ্রমাস গণনা করুন।* | ___ ___ lunar cycles after EID  ঈদের থথথথ চন্দ্রমাস পর  88. Refused to answer উত্তর দিতে অস্বীকার  99. Don’t know জানি না |  |
| 207 | Who takes the decision to vaccinate or not vaccinate this child?  আপনার শিশুর টিকা দেয়া / না দেয়া সিদ্ধান্ত কে নেয় ?  *Do not reach choices. Mark all that are mentioned.*  পছন্দগুলো বলবেন না। *যাদের নাম বলা হয় সবগুলো লিপিবদ্ধ করুন* ন। | 1. Mother মা  2. Father বাবা  3. Sister বোন  4. Brother ভাই  5. Other female relative অন্য কোন মহিলা আত্মীয়  6. Other male relative অন্য কোন পুরুষ আত্মীয়  7. Other অন্যান্য  8. The child by himself or herself শিশু নিজেই  9. No one কেউ না  88. Refused to answer উত্তর দিতে অস্বীকার  99. Don’t know জানি না |  |
| *If card is available, use date on vaccination card to answer questions. If no date is written on the card(s) or if card is missing, ask corresponding questions. For these questions, it may be helpful to use the event of Kurbani Eid, 01 Sep 2017, (or their arrival date if close to Kurbani Eid) to associate the first 2 campaigns (MR 1^st^ round and OCV 1^st^ round). As a reference, children under 2 years of age should have received injections in the thigh. Children 2 years and older should have received injections in the arm.*  যদি কার্ড পাওয়া যায় তবে প্রশ্নগুলির উত্তর দেওয়ার জন্য টিকা কার্ডের তারিখ ব্যবহার করুন। যদি কোনও কার্ডে তারিখ লেখা না থাকে বা যদি কার্ড হারিয়ে যায়, তাহলে সংশ্লিষ্ট প্রশ্নগুলি জিজ্ঞাসা করুন।  এই প্রশ্নগুলি প্রথম দুটো টিকাদান ক্যাম্পেইন (এমআর ১ম রাউন্ড এবং ওসিভি ১ম রাউন্ড) এর সাথে সংযুক্ত করার জন্য কোরবানি ঈদ ০১ সেপ্টেম্বর ২০১৭, (অথবা তাদের আগমনের তারিখ যদি কোরবানি ঈদের কাছাকাছি হয়) ব্যবহার করা যেতে পারে।  একটি রেফারেন্স হিসাবে বলা যায়, ২ বছরের কম বয়সী শিশুদের ঊরুতে এবং ২ বছর এবং তার উপরের বয়সের শিশুদের বাহুতে ইনজেকশন পাওয়া উচিত। | | | |
|  | How many times did this child take diphtheria vaccine?  এই শিশুটি কতবার ডিপথেরিয়ার টিকা নিয়েছিল?  *This question is asked to guide the next few questions and will not be recorded.*  পরবর্তী কয়েকটি প্রশ্নের দিক নির্দেশনার এই প্রশ্নটি করা হবে কিন্তু রেকর্ড করা হবে না। |  |  |
| 208 | About a month ago, there was a diphtheria vaccination campaign that included **one diphtheria injection** for older children. Did this child receive the injection? This child might have had **pain on touch** and maybe slight fever, which is normal. You may have received a **vaccination card** as well.  প্রায় এক মাস আগে, একটি ডিপথেরিয়া টিকাদান অভিযান হয়েছিল, যেখানে একটি ডিপথেরিয়া ইনজেকশন বয়স্ক শিশুদের দেয়া হয়েছিল।  আপনার শিশুটি কি সেখানে একটি ডিপথেরিয়া ইনজেকশন দেয়া হয়েছিল?  টিকাদান পরবর্তী সময়ে, সামান্য ব্যাথা অথবা সামান্য জ্বর হতে পারে, যা স্বাভাবিক। আপনি সেখানে একটি টিকাদান কার্ডও পেতে পারেন  *(10–29 March 2018:*  *Td, 2^nd^ round for 7years–<15years)*  (মার্চ, ২০১৮: টিটেনাস-ডিপথেরিয়া ২য় রাউন্ড- ৭-১৫ বছর বয়সী শিশুর জন্য) | 1. Yes, confirmed by date in vaccination card --->  হ্যাঁ, টিকা কার্ডের তারিখ অনুযায়ী নিশ্চিত  2. Yes, by recall ----------------------------------->  হ্যাঁ,স্মরন দ্বারা  3. No না  88. Refused to answer উত্তর দিতে রাজি না ------>  99. Don’t know জানি না -------------------------> | Q307  Q307  Q309  Q309 |
| 209 | What are the reasons why the child did not get vaccinated?  শিশুটির টিকা না পাওয়ার কারণগুলো কি ?  *Instructions:*  *The respondent should be prompted: ‘Are there any other reasons?’ until they have mentioned all reasons.*  *Do not read choices. Mark all that are mentioned.*  নির্দেশাবলী:  উত্তরদাতাকে অনুরোধ করা উচিত: 'অন্য কোন কারণ আছে কি না?'  পছন্দগুলি পড়বেন না । সেগুলো উল্লেখ করবে সেসব চিহ্নিত করুন। | 1. Caregiver was unaware of the vaccination campaign  পরিচর্যাকারি টিকাদান ক্যাম্পেইন সম্পর্কে সচেতন ছিলনা  2. Caregiver thinks that child received all required vaccines  পরিচর্যাকারি মনে করে যে শিশুটি সব প্রয়োজনীয় টিকা গ্রহন করেছে  3. Caregiver does not think vaccines are necessary  পরিচর্যাকারি চান না যে শিশুটি টিকা গ্রহন করুক  4. Caregiver is fearful of vaccines or fearful of multiple injections  পরিচর্যাকারী অনেকগুলো ইনজেকশন এক সঙ্গে দিতে হবে, তাই ভয় পেয়েছিলেন  5. Caregiver or child is fearful about not going to heaven or being converted to Christianity  পরিচর্যাকারী জান্নাতে না যেতে পারা অথবা খ্রীস্ট ধর্মে রূপান্তরিত হওয়ার ভয় পেয়েছেন  6. Female caregiver or child not comfortable leaving the house  মহিলা পরিচর্যাকারী অথবা শিশুটির পক্ষে ঘরটি ছেড়ে যাওয়া সম্ভবপর ছিল না  7. Female caregiver or child not comfortable taking vaccine from a male vaccinator  মহিলা পরিচর্যাকারী অথবা শিশুটির নিকট পুরুষ টিকাদানকারী গ্রহনযোগ্য ছিল না  8. The child got fever/swelling after last vaccination  শিশুটির শেষ টিকা গ্রহনের পর জ্বর / টিকার স্থানের ফুলে গিয়েছিল  9. No one was available to take the child to vaccination.  শিশুকে টিকা দিতে নিয়ে যাওয়ার জন্য কেউ ছিলনা  10. Child was not available (e.g., not at home, sick) at time of vaccination  শিশুটি বাসায় ছিল না / অসুস্থ ছিল  11. Child was afraid of needles/pain  শিশুটি ইনজেকশন/ ব্যাথার ভয় পেয়েছিল  12. Father or head of household does not allow  শিশুর পিতা / পরিবার প্রধান অনুমতি দেয়নি  13. Vaccinator treated caregiver or child poorly  টিকাদান কারী শিশু/ পরিচর্যাকারীর সাথে ভালো ব্যবহার করেননি  14. Other অন্যান্য  88. Refused to answer উত্তর দিতে অস্বীকার  99. Don’t know জানি না | Q309 |
| 210 | Who took the child to get vaccinated?  টিকা দিতে কে শিশুকে নিয়ে গিয়েছিল?  *Do not reach choices. Mark only one.*  পছন্দগুলো পড়িবেন না, শুধুমাত্র একটিতে টিক চিহ্ন দিন। | 1. Mother মা  2. Father বাবা  3. Sister বোন  4. Brother fvB  5. Other female relative অন্য কোন মহিলা আত্মীয়  6. Other male relative অন্য কোন পুরুষ আত্মীয়  7. Neighbor/friend প্রতিবেশী / বন্ধু  8. Social mobilizer সমাজসেবী  9. Vaccinated at school স্কুলে টিকা দেয়া হয়েছিল  10. Vaccinated at madrassa মাদ্রাসায় টিকা দেয়া হয়েছিল  11. Child went by him/her self শিশু নিজে গিয়েছে  12. Other অন্যান্য ---------------------  88. Refused to answer উত্তর দিতে অস্বীকার  99. Don’t know জানি না |  |
| 211 | Before the last campaign, there was a diphtheria vaccination campaign that included **one diphtheria injection** for older children. Did this child receive the injection? This child might have had **pain on touch** and maybe slight fever, which is normal. You may have received a **vaccination card** as well.  গত টিকাদান অভিযান এর আগে আরও একটি ডিপথেরিয়া টিকাদান অভিযান হয়েছিল, যেখানে বয়স্ক শিশুদের একটি ডিপথেরিয়া ইনজেকশন দেয়া হয়েছিল।  আপনার শিশুটি কি সেখানে একটি ডিপথেরিয়া ইনজেকশন পেয়েছিল্ ?  টিকাদান পরবর্তী সময়ে, সামান্য ব্যাথা অথবা সামান্য জ্বর হতে পারে, যা স্বাভাবিক। আপনি সেখানে একটি টিকাদান কার্ডও পেতে পারেন  *(27 January–10 February 2018:*  *Td, 2^nd^ round for 7years–<15years)*  (২৭ জানুয়ারী থেকে ১০ ফেব্রুয়ারী, ২০১৮: ডিপথেরিয়া-টিটেনাস ২য় রাউন্ড- ৭ - ১৫ বছর বয়সী শিশুর জন্য) | 1. Yes, confirmed by date in vaccination card  হ্যাঁ, টিকা কার্ডের তারিখ অনুযায়ী নিশ্চিত  2. Yes, by recall হ্যাঁ,স্মরন দ্বারা  3. No না  88. Refused to answer উত্তর দিতে রাজি না  99. Don’t know জানি না |  |
| 212 | And before that campaign, there was another diphtheria vaccination campaign that included **one diphtheria injection** for older children. Did this child receive the injection? This child might have had **pain on touch** and maybe slight fever, which is normal. You may have received a **vaccination card** as well.  এবং সইে টিকাদান অভিযান এর আগে আরও একটি ডিপথেরিয়া টিকাদান অভিযান হয়েছিল, যেখানে বয়স্ক শিশুদের একটি ডিপথেরিয়া ইনজেকশন দেয়া হয়েছিল।  আপনার শিশুটি কি সেখানে একটি ডিপথেরিয়া ইনজেকশন পেয়েছিল্ ?  টিকাদান পরবর্তী সময়ে, সামান্য ব্যাথা অথবা সামান্য জ্বর হতে পারে, যা স্বাভাবিক। আপনি সেখানে একটি টিকাদান কার্ডও পেতে পারেন  (২৭ জানুয়ারী থেকে ১০ ফেব্রুয়ারী, ২০১৮: ডিপথেরিয়া-টিটেনাস ১ম রাউন্ড- ৭ - ১৫ বছর বয়সী শিশুর জন্য)  *(12–31 December 2017:*  *Td, 1^st^ round for 7years–<15years)*  (১২-৩১ ডিসেম্বর, ২০১৭: ডিপথেরিয়া-টিটেনাস ১ম রাউন্ড  ৭ - ১৫ বছর বয়সী শিশুর জন্য) | 1. Yes, confirmed by date in vaccination card  হ্যাঁ, টিকা কার্ডের তারিখ অনুযায়ী নিশ্চিত  2. Yes, by recall হ্যাঁ,স্মরন দ্বারা  3. No না  88. Refused to answer উত্তর দিতে রাজি না  99. Don’t know জানি না |  |
|  | How many times did this child take measles vaccine?  এই শিশুটি কতবার হামের টিকা নিয়েছে?  *This question is asked to guide the next few questions and will not be recorded.*  এই প্রশ্নটি পরবর্তী কয়েকটি প্রশ্নের দিক নির্দেশনার জন্য বলা হয়েছে এবং এটি রেকর্ড করা হবে না। |  |  |
| 213 | Before the diphtheria vaccination campaign, there was a vaccination campaign that included **one** **injection for measles and NO oral drops**. Did this child receive the injection? You may have received a **vaccination card** as well.  ডিপথেরিয়া টিকাদান অভিযান এর আগে আরও একটি অভিযান হয়েছিল, যেখানে একটি হামের টিকার ইনজেকশন দেয়া হয়েছিল, কোনো পোলিও ড্রপ মুখে খাওয়ানো হযনি।  আপনার শিশুটি কি টিকা পেয়েছিল?  আপনি সেখানে একটি টিকাদান কার্ডও পেতে পারেন  *(18 November–05 December 2017:*  *MR, 2^nd^ round for 6months–<15years)*  (১৮ নভেম্বর থেকে ৫ ডিসেম্বর, ২০১৭ এম আর ২য় রাউন্ড-  ৬মাস - ১৫ বছর বয়সী শিশুর জন্য) | 1. Yes, confirmed by date in vaccination card  হ্যাঁ, টিকা কার্ডের তারিখ অনুযায়ী নিশ্চিত  2. Yes, by recall হ্যাঁ,স্মরন দ্বারা  3. No না  88. Refused to answer উত্তর দিতে রাজি না  99. Don’t know জানি না |  |
| 214 | There was another measles vaccination campaign a few weeks after Kurbani Eid that included cutting of the **Vitamin A red pill** and oral polio drops for young children and **one measles injection** for older children. Did this child receive the measles injection? You may have received a **vaccination card** as well.  কুরবানির ঈদের কয়েক সপ্তাহ পরে আরও একটি হামের টিকাদান অভিযান হয়েছিল, যেখানে একটি ভিটামিন এ এর লাল পিল, একটি হামের টিকার ইনজেকশন দেয়া হয়েছিল ও পোলিও ড্রপ মুখে খাওয়ানো হয়েছিল ।  আপনার শিশুটি কি টিকাগুলো পেয়েছিল ?  আপনি সেখানে একটি টিকাদান কার্ডও পেতে পারেন  *(16 September–03 October 2017:*  *OPV only 0–<6months*  *OR MR+OPV+Vitamin A, 1^st^ round for 6months–<5years*  *OR MR only for 5years–<15years)*  (১৬ সেপ্টেম্বর থেকে ০৩ অক্টোবর, ২০১৭:  ওপিভি-০ থেকে অনুর্ধ্ব ৬ মাস  ভিটামির এ+ এম আর + ওপিভি- ৬ মাস- অনুর্ধ্ব ৫ বছর বয়সী শিশুর জন্য  এম আর ১ম রাউন্ড ৫ বছর -অনুর্ধ্ব ১৫ বছর বয়সী শিশুর জন্য) | 1. Yes, confirmed by date in vaccination card  হ্যাঁ, টিকা কার্ডের তারিখ অনুযায়ী নিশ্চিত  2. Yes, by recall হ্যাঁ,স্মরন দ্বারা  3. No না  88. Refused to answer উত্তর দিতে রাজি না  99. Don’t know জানি না |  |
| 215 | After the Vitamin A / measles campaign, there was a vaccination campaign that included **oral cholera vaccine in a small bottle for both adults and children** (from 1 year and above). Did this child receive the oral vaccine? You may have received **soap** as well.  ভিটামিন এ /হামের টিকাদান ক্যাম্পেইনের পরে, আরেকটি টিকাদান ক্যাম্পেইন হয়েছিল যার মধ্যে অন্তর্ভুক্ত ছিল একটি ছোট বোতল যা ছিল বয়স্ক ও শিশুদের উভয়ের জন্য (১বছর এবং তারও বেশি)। এই শিশুটিকে কি মৌখিক টিকা দেওয়া হয়েছিল? আপনি একটি সাবান ও পেয়ে থাকতে পারেন।  *(10–18 October 2017:*  *OCV, 1^st^ round for ≥1year)*  (১০-১৮ অক্টোবর ২০১৭: ওসিভি প্রথম রাউন্ড) > ১ বছর) | 1. Yes হ্যাঁ  2. No না  88. Refused to answer উত্তর দিতে রাজি না  99. Don’t know জানি না |  |
| 216 | When you and your family entered Bangladesh, did this child take any vaccination at the border, either injections or oral polio drops?  যখন আপনি এবং আপনার পরিবার বাংলাদেশে ঢুকলেন, তখন এই শিশুটিকে কি সীমান্তে কোনও টিকা - ইনজেকশন বা মৌখিক পোলিও ড্রপ দেওয়া হয়েছিল? | 1. Yes, confirmed by date in vaccination card  হ্যাঁ, টিকা কার্ডের তারিখ অনুযায়ী নিশ্চিত  2. Yes, by recall হ্যাঁ,স্মরন দ্বারা  3. No না  88. Refused to answer উত্তর দিতে রাজি না  99. Don’t know জানি না |  |
| 217 | Can you share any additional vaccination cards or medical records related to this child?  আপনি কি এই শিশুর সাথে সম্পর্কিত কোনও অতিরিক্ত টিকাদান কার্ড বা মেডিকেল রেকর্ড দেখাতে পারেন?  *If there are any additional vaccination cards that do not correspond to campaign doses, vaccination at the border or vaccination for being a diphtheria contact, indicate which vaccines were received.*  যদি কোনো অতিরিক্ত টিকাদান কার্ড থাকে যা ক্যাম্পেইন ডোজ, সীমান্তে টিকা বা ডিপথেরিয়ার কারনে টিকার সাথে সম্পর্কিত নয় তাহলে, কোন টিকা তা নির্দেশ করুন। | 1. OPV, confirmed by date in vaccination card  ওপিভি, টিকা কার্ডের তারিখ অনুযায়ী নিশ্চিত  2. PCV, confirmed by date in vaccination card  পিসিভি টিকা কার্ডের তারিখ দ্বারা নিশ্চিত  3. Penta, confirmed by date in vaccination card  পেন্টা টিকা কার্ডের তারিখ অনুযায়ী নিশ্চিত  4. Td, confirmed by date in vaccination card  টিদি, টিকা কার্ডের তারিখ দ্বারা নিশ্চিত  5. MR, confirmed by date in vaccination card  এম.আর.টিকা কার্ডের তারিখ অনুযায়ী নিশ্চিত  6. BCG, confirmed by date in vaccination card  বিসিজি, টিকাদান কার্ড থেকে নিশ্চিত  7. IPV, confirmed by date in vaccination card  আইপিভি, টিকাদান কার্ড থেকে নিশ্চিত  8. Other অন্যান্য  9. No other vaccination card available  অন্য কোন টিকা কার্ড পাওয়া যায় না |  |
| 218 | When this child lived in Myanmar, did this child ever take polio vaccine or oral polio drops. Polio is a vaccine given by dropping liquid into a child’s mouth. Oral polio drops are different from Vitamin A drops, which usually are red or blue pills.  যখন এই শিশুটি মায়ানমারে বসবাস করত, তখন এই শিশুটি কি কখনো পোলিও টিকা বা মৌখিক পোলিও ড্রপ গ্রহন করেছে? পোলিও একটি তরল টিকা যা শিশুর মুখের মধ্যে ড্রপ দ্বারা দেওয়া হয়। | 1. Yes, confirmed by date in vaccination card  হ্যাঁ, টিকা কার্ডের তারিখ অনুযায়ী নিশ্চিত  2. Yes, by recall হ্যাঁ,স্মরন দ্বারা  3. No না  88. Refused to answer উত্তর দিতে রাজি না  99. Don’t know জানি না |  |
| 219 | When this child lived in Myanmar, did this child ever take any vaccination through injection?  যখন এই শিশু মায়ানমারে বসবাস করত, তখন কি এই শিশুটি ইনজেকশন এর মাধ্যমে কোনো টিকা নিয়েছে ? | 1. Yes, confirmed by date in vaccination card  হ্যাঁ, টিকা কার্ডের তারিখ অনুযায়ী নিশ্চিত  2. Yes, by recall হ্যাঁ,স্মরন দ্বারা  3. No না  88. Refused to answer উত্তর দিতে রাজি না  99. Don’t know জানি না |  |
| 220 | *Does the child have a BCG scar? Check the child’s upper right and left arms. (Do not ask the caregiver.)*  এই শিশুটির কি বিসিজি স্কার আছে? শিশুর ডান এবং বাম বাহুর উপরে পরীক্ষা করুন। (পরিচর্যাকারিকে জিজ্ঞাসা করবেন না।) | 1. Yes হ্যাঁ  2. No না  3. Not sure if scar is from BCG  নিশ্চিত না স্কারটি বিসিজির কিনা  99. Child not available শিশুটি উপস্থিত নাই |  |
| 221 | In the past two weeks, has this child had a fever without rash?  বিগত ২ সপ্তাহে কি শিশুর র‌্যাশ ছাড়া জ্বর হয়েছিলো ? | 1. Yes হ্যাঁ  2. No না  88. Refused to answer উত্তর দিতে রাজি না  99. Don’t know জানি না |  |
| 222 | After arrival in Bangladesh, has this child had measles?  বাংলাদেশে আগমনের পর, শিশুর কি কখনো হাম হয়েছিলো ? | 1. Yes, confirmed by health facility document  হ্যাঁ, স্বাস্থ্য কেন্দ্রের দলিল অনুযায়ী নিশ্চিত  2. Yes, verbal report that the child was diagnosed at a clinic  হ্যাঁ, কোনো ক্লিনিকে মৌখিকভাবে নিশ্চিত  3. Yes, verbal report that the child was diagnosed by a local healer  হ্যাঁ, কোনো স্থানীয় পল্লী চিকিসক কর্তৃক মৌখিকভাবে নিশ্চিত  4. Yes, verbal report but did not seek diagnosis  হ্যাঁ, মৌখিক রিপোর্ট কিন্তু ওই রোগের জন্য যায়নি  5. No না  88. Refused to answer উত্তর দিতে রাজি না  99. Don’t know জানি না |  |
| 223 | After arrival in Bangladesh, has this child had diphtheria?  বাংলাদেশে আগমনের পর, শিশুর কি কখনো ডিপথেরিয়া হয়েছিলো ? | 1. Yes, confirmed by health facility document  হ্যাঁ, স্বাস্থ্য কেন্দ্রের দলিল অনুযায়ী নিশ্চিত  2. Yes, verbal report that the child was diagnosed at a clinic  হ্যাঁ, কোনো ক্লিনিকে মৌখিকভাবে নিশ্চিত  3. Yes, verbal report that the child was diagnosed by a local healer  হ্যাঁ, কোনো স্থানীয় পল্লী চিকিসক কর্তৃক মৌখিকভাবে নিশ্চিত  4. Yes, verbal report but did not seek diagnosis  হ্যাঁ, মৌখিক রিপোর্ট কিন্তু ওই রোগের ক্লিনিকে জন্য যায়নি  5. No না  88. Refused to answer উত্তর দিতে রাজি না  99. Don’t know জানি না |  |

**END OF QUESTIONNAIRE**

**ধন্যবাদ জানিয়ে সাক্ষাতকারটি শেষ করুন।**

**INSERT Bangla of above end instruction.**
